# HIV self-testing alone or with additional interventions, including financial incentives, and linkage to care or prevention among male partners of antenatal care clinic attendees in Malawi: An adaptive multi-arm, multi-stage cluster randomised trial

**DOI:** 10.1371/journal.pmed.1002719

**Published:** 2019-01-02

**Authors:** Augustine T. Choko, Elizabeth L. Corbett, Nigel Stallard, Hendramoorthy Maheswaran, Aurelia Lepine, Cheryl C. Johnson, Doreen Sakala, Thokozani Kalua, Moses Kumwenda, Richard Hayes, Katherine Fielding

**Affiliations:** 1 TB/HIV Group, Malawi–Liverpool–Wellcome Clinical Research Programme, Blantyre, Malawi; 2 Department of Infectious Disease Epidemiology, London School of Hygiene & Tropical Medicine, London, United Kingdom; 3 Department of Clinical Research, London School of Hygiene & Tropical Medicine, London, United Kingdom; 4 Warwick Medical School, University of Warwick, Coventry, United Kingdom; 5 Institute of Psychology, Health and Society, University of Liverpool, Liverpool, United Kingdom; 6 Department of Global Health and Development, London School of Hygiene & Tropical Medicine, London, United Kingdom; 7 World Health Organization, Geneva, Switzerland; 8 Department of HIV/AIDS, Ministry of Health, Lilongwe, Malawi; University of California, San Francisco, UNITED STATES

## Abstract

**Background:**

Conventional HIV testing services have been less comprehensive in reaching men than in reaching women globally, but HIV self-testing (HIVST) appears to be an acceptable alternative. Measurement of linkage to post-test services following HIVST remains the biggest challenge, yet is the biggest driver of cost-effectiveness. We investigated the impact of HIVST alone or with additional interventions on the uptake of testing and linkage to care or prevention among male partners of antenatal care clinic attendees in a novel adaptive trial.

**Methods and findings:**

An adaptive multi-arm, 2-stage cluster randomised trial was conducted between 8 August 2016 and 30 June 2017, with antenatal care clinic (ANC) days (i.e., clusters of women attending on a single day) as the unit of randomisation. Recruitment was from Ndirande, Bangwe, and Zingwangwa primary health clinics in urban Blantyre, Malawi. Women attending an ANC for the first time for their current pregnancy (regardless of trimester), 18 years and older, with a primary male partner not known to be on ART were enrolled in the trial after giving consent. Randomisation was to either the standard of care (SOC; with a clinic invitation letter to the male partner) or 1 of 5 intervention arms: the first arm provided women with 2 HIVST kits for their partners; the second and third arms provided 2 HIVST kits along with a conditional fixed financial incentive of $3 or $10; the fourth arm provided 2 HIVST kits and a 10% chance of receiving $30 in a lottery; and the fifth arm provided 2 HIVST kits and a phone call reminder for the women’s partners. The primary outcome was the proportion of male partners who were reported to have tested for HIV and linked into care or prevention within 28 days, with referral for antiretroviral therapy (ART) or circumcision accordingly. Women were interviewed at 28 days about partner testing and adverse events. Cluster-level summaries compared each intervention versus SOC using eligible women as the denominator (intention-to-treat). Risk ratios were adjusted for male partner testing history and recruitment clinic. A total of 2,349/3,137 (74.9%) women participated (71 ANC days), with a mean age of 24.8 years (SD: 5.4). The majority (2,201/2,233; 98.6%) of women were married, 254/2,107 (12.3%) were unable to read and write, and 1,505/2,247 (67.0%) were not employed. The mean age for male partners was 29.6 years (SD: 7.5), only 88/2,200 (4.0%) were unemployed, and 966/2,210 (43.7%) had never tested for HIV before. Women in the SOC arm reported that 17.4% (71/408) of their partners tested for HIV, whereas a much higher proportion of partners were reported to have tested for HIV in all intervention arms (87.0%–95.4%, *p* < 0.001 in all 5 intervention arms). As compared with those who tested in the SOC arm (geometric mean 13.0%), higher proportions of partners met the primary endpoint in the HIVST + $3 (geometric mean 40.9%, adjusted risk ratio [aRR] 3.01 [95% CI 1.63–5.57], *p* < 0.001), HIVST + $10 (51.7%, aRR 3.72 [95% CI 1.85–7.48], *p* < 0.001), and phone reminder (22.3%, aRR 1.58 [95% CI 1.07–2.33], *p* = 0.021) arms. In contrast, there was no significant increase in partners meeting the primary endpoint in the HIVST alone (geometric mean 17.5%, aRR 1.45 [95% CI 0.99–2.13], *p* = 0.130) or lottery (18.6%, aRR 1.43 [95% CI 0.96–2.13], *p* = 0.211) arms. The lottery arm was dropped at interim analysis. Overall, 46 male partners were confirmed to be HIV positive, 42 (91.3%) of whom initiated ART within 28 days; 222 tested HIV negative and were not already circumcised, of whom 135 (60.8%) were circumcised as part of the trial. No serious adverse events were reported. Costs per male partner who attended the clinic with a confirmed HIV test result were $23.73 and $28.08 for the HIVST + $3 and HIVST + $10 arms, respectively. Notable limitations of the trial included the relatively small number of clusters randomised to each arm, proxy reporting of the male partner testing outcome, and being unable to evaluate retention in care.

**Conclusions:**

In this study, the odds of men’s linkage to care or prevention increased substantially using conditional fixed financial incentives plus partner-delivered HIVST; combinations were potentially affordable.

**Trial registration:**

ISRCTN 18421340.

## Abstract 2

In a cluster-randomized trial, Augustine Choko and colleagues study the effects of incentives for HIV self-testing on partners of antenatal care attendees.

## Author summary

**Why was this study done?:**

- Men are underserved by current HIV testing services in settings with high HIV prevalence and thus remain a priority target population for many HIV policy makers.
- The aim of the study was to identify interventions that could increase male partner testing and subsequent linkage to care or prevention through providing oral HIV self-test kits via a pregnant partner attending an antenatal care clinic (ANC).

**What did the researchers do and find?:**

- We conducted a cluster randomised study in urban Blantyre, Malawi, recruiting pregnant women attending an ANC for the first time for their current pregnancy.
- We assessed the impact of providing oral self-test kits to male partners via the ANC attendees on male partner testing and clinic attendance for post-test services within 28 days of the woman’s enrolment. Additionally, we offered a guaranteed monetary financial incentive of $3 or $10 or a chance to win $30 in a lottery to male partners, conditional on clinic attendance. We also tested the impact of phone call reminders to the male partner immediately after the woman collected the kits, repeated after 5 days.
- A total of 2,349 eligible pregnant women were recruited to the study. Compared to a standard of care (SOC) of a personalised invitation letter to the male partner to attend the clinic for HIV testing and to receive post-test services, male partner testing increased from 17% with SOC to between 87% and 95% with the oral self-test kits, as reported by the women.
- Subsequent male partner clinic attendance within 28 days of the woman’s enrolment was 13.0% with SOC; 17.5% with self-test kits only; 40.9% and 51.7% with self-test kits plus $3 and $10 financial incentives, respectively; 18.6% with self-test kits plus lottery; and 22.3% with self-test kits plus phone reminder. No serious adverse events were reported either by the women or their male partners.

**What do these findings mean?:**

- We found that providing oral self-test kits to men via a pregnant partner who was attending a clinic for antenatal care services increased male partner HIV testing.
- Additional interventions such as financial incentives may encourage men to attend the clinic for post-test HIV services alongside partner-delivered self-test kits.

## Introduction

In 2016, 1.0 million people died of diseases associated with HIV infection, and 1.8 million were newly infected [1]. Eastern and southern Africa have been disproportionately affected by the epidemic, and face challenges in reaching men: regionally, only 52% of men living with HIV are aware of their infection [2], whilst deaths from AIDS-related illnesses are 27% higher amongst men than women [1]. Achieving the 2020 UN targets of having 90% of all people living with HIV diagnosed, 90% of those diagnosed on antiretroviral therapy (ART), and 90% of those on treatment virally suppressed [1] should bring major reductions in HIV incidence and mortality, but may also require novel service delivery approaches [1].

HIV self-testing (HIVST), whereby individuals collect their own sample (oral or blood), conduct the test, and interpret their result [3], has been found to be highly acceptable to those wishing to test for HIV infection and has been shown to increase coverage and frequency of testing in high-risk men [4]. In Malawi, community-based distribution of HIVST kits plus home-based ART initiation in an urban slum setting increased demand for ART, and was shown to be cost-effective [5,6]. In Kenya, secondary distribution of HIVST kits by antenatal care clinic (ANC) attendees to their male partners increased uptake of HIV testing, although subsequent uptake of post-test HIV care and prevention services (“linkage”) was not optimally measured [7]. Linkage after HIVST has been previously evaluated from the perspective of delivery of ART [6,8,9], with mixed results. Demand for ART increased 3-fold when community participants who self-tested positive were offered 2 weeks of ART at home in Malawi [6], while self-testing did not increase ART initiation in a study among female sex workers in Zambia [8]. Studies that assess linkage after HIVST to HIV prevention services such as circumcision or pre-exposure prophylaxis (PrEP) are very rare.

Antenatal services in eastern and southern Africa have achieved near-universal HIV testing amongst pregnant women [10], providing an ideal opportunity for engaging male partners and extending benefits to the unborn child [11]. We used a novel multi-arm, multi-stage (MAMS) cluster randomised trial design [12] to evaluate the efficacy, safety, and costs of a number of candidate interventions including HIVST alone and in combination with conditional fixed financial incentives, lotteries, or phone reminders.

## Methods

### Design, setting, and participants

This was a 2-stage Phase II MAMS adaptive cluster randomised trial conducted at 3 primary health clinics in Blantyre, Malawi, between 8 August 2016 and 30 June 2017 [12] (S1 Text). We block randomised ANC days (representing clusters of women) to 1 of 6 trial arms (standard of care [SOC] and 5 intervention arms; ratio 1:1:1:1:1:1) in stage 1. All women were screened for eligibility at their routine ANC visit, with each ANC day offering services to first time attendees eligible as a cluster. Those eligible (first ANC visit for current pregnancy, residence in Blantyre, with a male partner not known to be on ART) and who provided informed consent were allocated to the study arm assigned to that day. At the end of stage 1, intervention arms could be dropped if *p* > 0.2 compared to SOC, or for safety concerns including intimate partner violence, guided by an independent data and safety monitoring board (DSMB). Further ANC days, as determined to be needed by sample size re-calculation, were then randomised in equal proportions to the remaining trial arms for stage 2.

### Interventions

An independent statistician generated the allocation sequence for the trial arms. The arm assignment of each ANC day was only revealed to the field team on the morning of the recruitment day, to enhance allocation concealment. In the SOC arm, women were given an invitation letter addressed to their male partner informing them of the importance of having an HIV test, and the availability of HIV testing and fast track referral for HIV treatment or voluntary medical male circumcision (VMMC) services through a male-friendly clinic (MFC) set up within the recruiting facility [13]. The male partner had to present the letter to identify himself and his partner at the clinic in order to access MFC services.

Treatment for participants in all 5 intervention arms included the SOC letter and clinic access together with 2 prequalified oral HIVST kits (OraQuick HIV Self Test, OraSure Technologies, Bethlehem, PA, US) for the woman to take home for her male partner: 1 arm offered this combination only (“ST”). Two arms offered an additional conditional fixed cash financial incentive of $3 or $10 (referred to as “ST + $3” and “ST + $10”, respectively), and a fourth intervention arm offered a 10% chance of winning $30 (“ST + lottery”). The fifth intervention arm included a phone call to the male partner on the day the woman enrolled, which was repeated 5 days later if partner had not come to the clinic (“ST + reminder”). Regardless of HIV self-test results, the male partners were asked to present to the clinic with their letter as identification. Incentives were given to the men and were conditional on their attending clinic-based HIV treatment or prevention services within 28 days. The study’s selection of $3, $10, and a phone reminder as additional interventions used formative study results [13]. The $3 amount reflected the out-of-pocket costs of accessing facility HIV testing services in this setting [14].

### Outcomes and measurement

The primary outcome was the proportion of male partners who tested for HIV and linked into care or prevention within 28 days of the woman’s enrolment, assuming 1 man per enrolled woman. All men in the HIVST arms were asked to present their used HIVST kit, and could repeat HIVST with a study HIV counsellor at the MFC. In the SOC arm, HIV testing used finger-prick rapid diagnostic test kits following Malawi national HIV testing guidelines, provided to male partners who attended the MFC.

Confirmatory testing followed Malawi national guidelines and was conducted in all trial arms by Ministry of Health HIV counsellors at the clinic. There was referral for ART if the male partner was HIV positive, for VMMC if the male partner was HIV negative and uncircumcised, or for counselling if the male partner was HIV negative and already circumcised. We conducted 2 post hoc analyses. First, we restricted the numerator to confirmed HIV positive men referred for ART and HIV negative men referred for VMMC, thus excluding men who were HIV negative and already circumcised. Second, the numerator was restricted to men who started ART or got circumcised.

The secondary outcomes were the proportion of male partners who tested for HIV within 28 days, as reported by the women through audio computer-assisted self-interview (ACASI) at follow-up, and the risk of adverse events (AEs) and serious adverse events (SAEs) [12] within 28 days of the woman’s enrolment, as reported through ACASI.

### Analysis and sample size

Assuming each cluster enrolled 40–60 women, and that 25% of male partners achieved the primary outcome in the SOC arm with a coefficient of variation (*k*) of 0.10 [12], 6 clusters per arm in stage 1 and 7 clusters per arm in stage 2 gave 80% power to detect a 15% absolute difference in proportions at a 20% and 5% significance level for stage 1 and stage 2, respectively. The target sample size for stage 2 was re-calculated using the observed harmonic mean number of women per cluster and the proportion achieving the primary outcome. The intention-to-treat principle was followed, using the number of consented women as the denominator in each cluster.

Baseline characteristics of male partners, as reported by the women, were compared across trial arms. A planned interim analysis at the end of stage 1 was conducted in order to make pre-planned trial adaptations. We analysed combined stage 1 and 2 data by comparing each intervention arm to the SOC arm and computing a risk ratio (RR) [15] and 95% confidence interval (CI), using a cluster-level summaries approach [15] due to the small number of clusters per arm. Any baseline imbalance was adjusted for using a 2-step approach [15]: First, we used a logistic regression model with baseline covariates to obtain an expected outcome; second, the cluster-level ratio of observed:expected outcomes was compared by arm. A *t* test was used to compute a *p*-value for each comparison, followed by a Dunnett test to correct for multiple comparisons [16]. The statistical analysis approach is described in S1 Appendix, and we completed a CONSORT Checklist (S1 CONSORT Checklist).

### Cost analysis

For each trial arm we recorded all resources used by those randomised to that arm and then costed each resource use item to estimate the total costs. We divided the total costs by the total number of men who tested for HIV and attended an MFC to estimate the cost per male partner who tested for HIV and attended an MFC, and by the total number of men who started ART or were referred for VMMC to estimate the cost per male partner who tested for HIV and either started ART or was referred for VMMC (S2 Appendix). Costs were estimated from the health provider perspective and are presented in 2016 US dollars.

### Ethical considerations

The trial was approved by the University of Malawi College of Medicine Research Ethics Committee and by the London School of Hygiene & Tropical Medicine Ethics Committee. Women provided written or witnessed (with thumb print for those illiterate) informed consent before undergoing any trial procedures. Written consent for male partners was waived by the 2 ethics committees. The waiver of consent for male partners was granted on the basis that HIV testing is a SOC service, but the study provided information for the male partners to read and voluntarily decide to take part in the trial.

## Results

**Fig 1:**
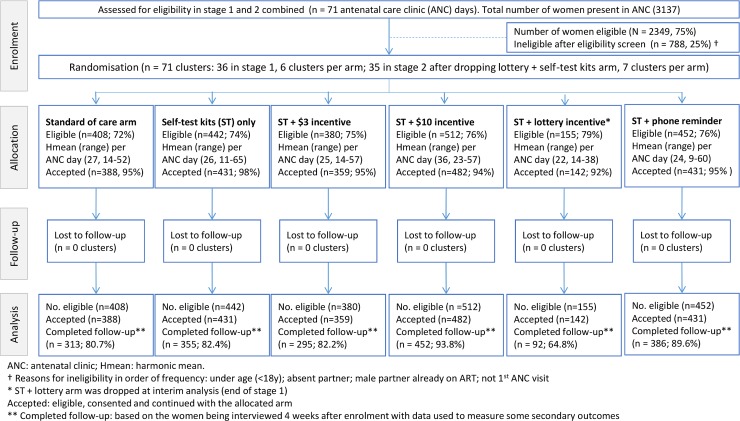
Consort flow diagram (stage 1 and 2 combined).

Recruitment and follow-up was completed between 8 August 2016 and 30 June 2017. In total, 3,137 pregnant women (71 clusters [ANC days]: 36 in stage 1 and 35 in stage 2) were screened, of whom 2,349 (74.9%) were eligible and consented (Fig 1). Baseline characteristics of male partners, as reported by the women, were reasonably balanced across trial arms except for male partner HIV testing history. Recruitment clinic was also imbalanced by trial arm (Table 1). All analyses were adjusted for these 2 covariates.

**Table 1:** Baseline characteristics of male partners as reported by women at enrolment (*n* = 2,349; stage 1 and 2 combined).

Variable	Characteristic	Trial arm					
		SOC	ST	ST + $3	ST + $10	ST + lottery*	ST + reminder
Eligible	*n*	408	442	380	512	155	452
Age (years)	Mean (SD)	29.95 (8.51)	29.58 (6.05)	28.89 (6.37)	29.31 (6.54)	30.30 (10.94)	29.31 (6.38)
Able to read and write	Yes	377 (97.2)	425 (98.6)	354 (98.6)	476 (98.8)	140 (98.6)	420 (97.4)
Level of education attained	Primary/no school	100 (25.8)	82 (19.1)	85 (23.6)	98 (20.3)	38 (26.8)	90 (20.9)
	Secondary, no MSCE	135 (34.8)	152 (35.3)	125 (34.8)	157 (32.6)	58 (40.8)	166 (38.5)
	Secondary, MSCE	127 (32.7)	157 (36.4)	121 (33.7)	169 (35.1)	36 (25.4)	142 (32.9)
	Any tertiary	26 (6.7)	40 (9.3)	28 (7.8)	58 (12.0)	10 (7.0)	33 (7.7)
Occupation	Paid employee	216 (55.7)	275 (63.8)	193 (53.8)	282 (58.5)	68 (47.9)	233 (54.1)
	Paid domestic worker	20 (5.2)	21 (4.9)	36 (10.0)	31 (6.4)	18 (12.7)	36 (8.4)
	Self-employed	130 (33.5)	111 (25.8)	108 (30.1)	123 (25.5)	43 (30.3)	135 (31.3)
	Unemployed	14 (3.6)	12 (2.8)	10 (2.8)	30 (6.2)	7 (4.9)	15 (3.5)
	Student	7 (1.8)	6 (1.4)	4 (1.1)	9 (1.9)	2 (1.4)	6 (1.4)
	Other	1 (0.3)	6 (1.4)	8 (2.2)	7 (1.5)	4 (2.8)	6 (1.4)
Ever tested for HIV	Never tested before	193 (49.7)	169 (39.2)	167 (46.5)	213 (44.2)	65 (45.8)	159 (36.9)
	Tested >12 months ago	98 (25.3)	140 (32.5)	101 (28.1)	153 (31.7)	49 (34.5)	170 (39.4)
	Tested ≤12 months ago	97 (25.0)	122 (28.3)	91 (25.3)	116 (24.1)	28 (19.7)	102 (23.7)
Recruitment PHC	Ndirande	73 (17.9)	67 (15.2)	98 (25.8)	99 (19.3)	42 (27.1)	66 (14.6)
	Bangwe	150 (36.8)	76 (17.2)	162 (42.6)	142 (27.7)	85 (54.8)	307 (67.9)
	Zingwangwa	185 (45.3)	299 (67.6)	120 (31.6)	271 (52.9)	28 (18.1)	79 (17.5)

Data are for all the male partners of eligible women, including those who discontinued participation after giving consent and learning trial arm.

*ST + lottery arm was dropped at interim analysis (end of stage 1).

MSCE, Malawi school certificate of education (4 years); PHC, primary health clinic; SD, standard deviation; SOC, standard of care; ST, self-test.

At interim analysis, the ST + lottery arm was dropped (*p* = 0.211; see S1 Table). Four intervention arms and the SOC arm continued to stage 2. The HIVST only arm was continued on the advice of the DSMB despite not meeting the relevant statistical criterion (*p* = 0.211; S1 Table); it is the a priori preferred option for policy makers in Malawi.

### Primary outcome

**Fig 2:**
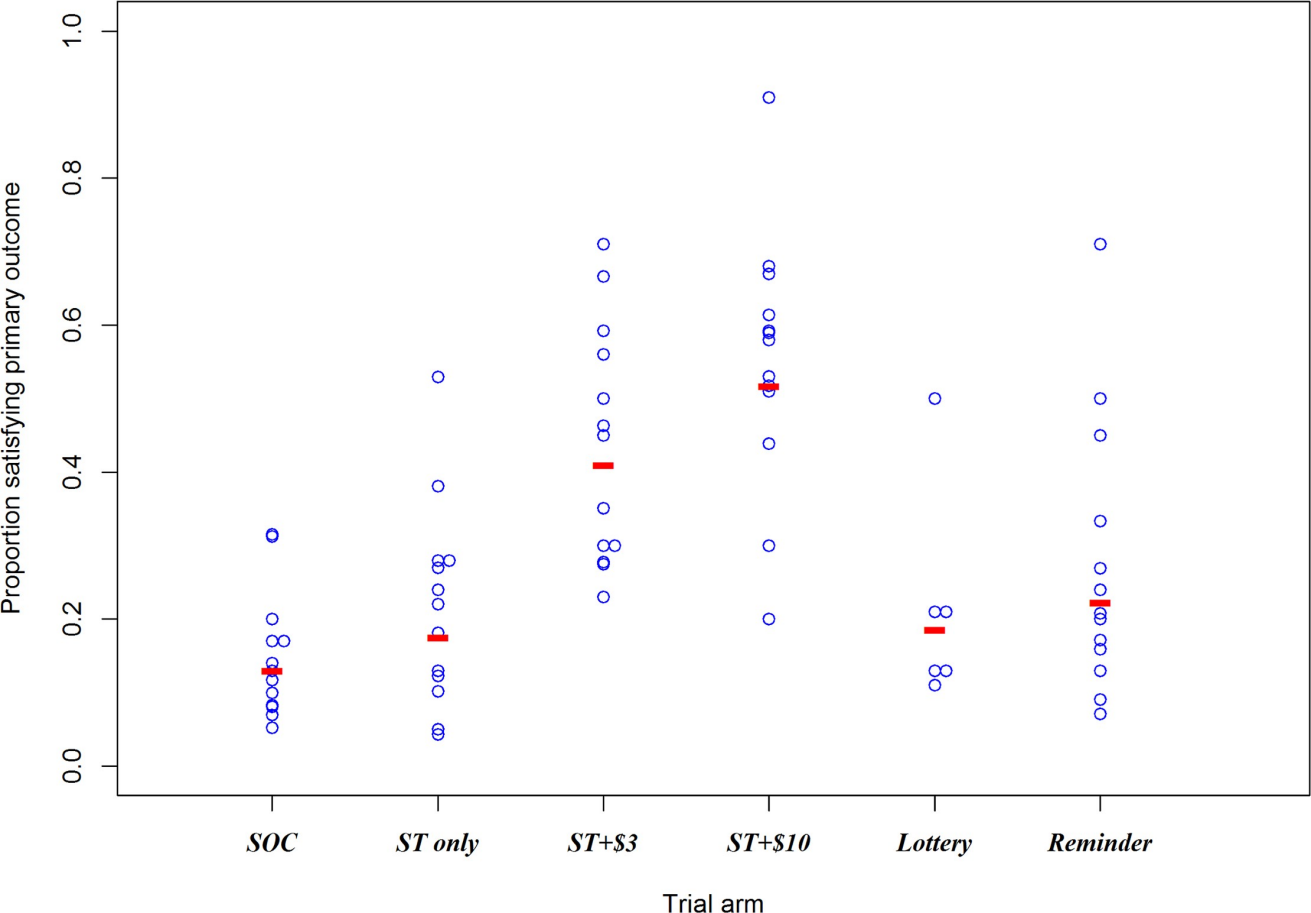
Cluster-level proportion for the primary outcome of evidence of testing and linking to MFC within 28 days, by trial arm. Lottery and reminder arms also had ST. Red horizontal line = geometric mean of cluster-level proportion. MFC, male-friendly clinic; SOC, standard of care; ST, self-test.

Overall, 676/2,349 (28.8%) male partners had an HIV test and attended the MFC within 28 days of the woman’s enrolment (Table 2). Compared to a geometric mean clinic attendance of 13.0% in the SOC arm, the ST only arm had a geometric mean of 17.5%, RR 1.35 (95% CI 0.90–2.01); the ST + $3 arm had a geometric mean of 40.9%, RR 3.15 (95% CI 2.11–4.70); the ST + $10 arm had a geometric mean of 51.7%, RR 3.98 (95% CI 2.66–5.95); the ST + lottery arm had a geometric mean of 18.6%, RR 1.43 (95% CI 0.96–2.13); and the ST + reminder arm had a geometric mean of 22.3%, RR 1.71 (95% CI 1.15–2.55) (Table 2; Fig 2). In adjusted analysis controlling for male partner testing history and recruitment clinic (*n* = 2,233), the estimates were as follows: adjusted RR (aRR) 1.45 (95% CI 0.99–2.13) for ST only, 3.01 (95% CI 1.63–5.57) for ST + $3, aRR 3.72 (95% CI 1.85–7.48) for ST + $10, aRR 1.53 (95% CI 0.93–2.52) for ST + lottery, and aRR 1.58 (95% CI 1.07–2.33) for ST + reminder (Table 2). Fidelity for the reminder intervention was good: nearly three-quarters of men received at least 1 phone call.

**Table 2:** Unadjusted and adjusted intervention effects by trial arm and trial stage for the primary outcome* and post hoc outcomes (stage 1 and 2 combined).

Outcome	Trial arm					
	SOC	ST only	ST + $3	ST + $10	ST + lottery^†^	ST + reminder^‡^
Number of clusters	13	13	13	13	6	13
Eligible women	408	442	380	512	155	452
Primary outcome*	56	85	155	266	30	84
Geometric mean	13.0%	17.5%	40.9%	51.7%	18.6%	22.3%
Risk difference	NA	4.5%	27.9%	38.7%	5.6%	9.3%
95% CI	NA	−0.3%; 9.3%	22.0%; 33.8%	33.2%; 44.1%	−1.3%; 12.5%	4.3%; 14.3%
Unadjusted RR	1	1.35	3.15	3.98	1.43	1.71
95% CI		0.90; 2.01	2.11; 4.70	2.66; 5.95	0.96; 2.13	1.15; 2.55
*p-*Value^§^	NA	0.332	<0.001	<0.001	0.332	0.079
Adjusted RR^#^	1	1.45	3.01	3.72	1.53	1.58
95% CI		0.99; 2.13	1.63; 5.57	1.85; 7.48	0.93; 2.52	1.07; 2.33
*p-*Value^§^	NA	0.130	<0.001	<0.001	0.211	0.021
***Outcomes at MFC***						
HIV-positive	3/56 (5.4%)	11/85 (12.9%)	11/155 (7.1%)	14/266 (5.3%)	4/30 (13.3%)	3/84 (3.6%)
Started ART	3/3 (100%)	10/11 (90.9%)	10/11 (90.9%)	13/14 (92.9%)	4/2 (100%)	2/3 (66.7%)
HIV-negative	53/56 (95.6%)	74/85 (87.1%)	144/155 (92.9%)	252/266 (94.7%)	26/30 (86.7%)	81/84 (96.4%)
Already circumcised	38/56 (67.9%)	43/85 (50.6%)	97/155 (62.6%)	168/266 (63.2%)	20/30 (66.7%)	42/84 (50.0%)
Uncircumcised	15/56 (26.8%)	31/85 (36.5%)	47/155 (30.3%)	84/266 (31.6%)	6/30 (20.0%)	39/84 (46.4%)
Circumcised	11/15 (73.3%)	17/31 (54.8%)	29/47 (61.7%)	55/84 (65.5%)	3/6 (50.0%)	20/39 (51.3%)
***Post-hoc outcomes***						
***Referred for ART or VMMC***	18/408 (4.4%)	42/442 (9.5%)	58/380 (15.3%)	98/512 (19.1%)	10/155 (6.5%)	42/452 (9.3%)
Risk difference	NA	5.1%	10.9%	14.7%	2.1%	4.9%
95% CI	NA	1.7%; 8.5%	6.8%; 15.0%	10.8%; 18.6%	−2.3%; 6.5%	1.6%; 8.2%
Unadjusted RR	1	1.64	3.45	3.38	1.37	1.87
95% CI		1.00; 2.68	2.10; 5.64	2.06; 5.53	0.83; 2.24	1.14; 3.07
Adjusted RR^#^	1	1.41	3.06	3.76	1.87	1.68
95% CI		0.79; 2.50	1.43; 6.57	1.76; 8.03	0.58; 6.01	0.90; 3.15
*p-*Value^§^	NA	0.088	<0.001	<0.001	0.297	0.104
***Started ART or were circumcised***	14/408 (3.4%)	27/442 (6.1%)	39/380 (10.3%)	68/444 (13.3%)	7/155 (4.5%)	22/452 (4.9%)
Risk difference	NA	2.7%	6.9%	9.9%	1.1%	1.5%
95% CI	NA	−0.1%; 5.5%	3.4%; 10.4%	6.5%; 13.3%	−2.6%; 4.8%	−1.2%; 4.1%
Adjusted RR^#^	1	2.30	4.53	4.40	2.91	2.81
95% CI		1.06; 5.00	1.01; 20.39	1.55; 12.48	0.53; 15.88	1.15; 6.88
*p-*Value^§^	NA	0.035	0.049	0.005	0.220	0.023

*Primary outcome: evidence of testing and linking to MFC within 28 days (regardless of HIV test result).

^†^10% chance of winning a prize equal to $30. This arm was dropped at interim analysis (end of stage 1).

^‡^Phone call.

^§^Adjusted for multiple comparisons using the Dunnett test.

^#^Adjusted for male partner history of HIV testing as reported by the woman at the recruitment clinic (*n* = 2,233).

ART, antiretroviral therapy; CI confidence interval; MFC male-friendly clinic; NA, not applicable; RR, risk ratio; SOC, standard of care; ST, self-test; VMMC, voluntary medical male circumcision.

Of the 676 men who attended the MFC, 46 (6.8%) had a newly confirmed HIV positive result, all of whom were referred for ART; 42 (91.3%) started ART the same day as their HIV diagnosis. Of the 630 (93.2%) HIV negative men, 408 (64.8%) were already circumcised. The remaining 222 (35.2%) uncircumcised men all agreed to referral for VMMC, with 135/222 (60.8%) undergoing the procedure within 28 days and a further 30 a month later. There was no evidence of clustering for the linkage outcome, with an intra-cluster correlation coefficient of 0.015 (95% CI 0.002–0.102, *p* = 0.082).

### Post hoc outcomes

**Fig 3:**
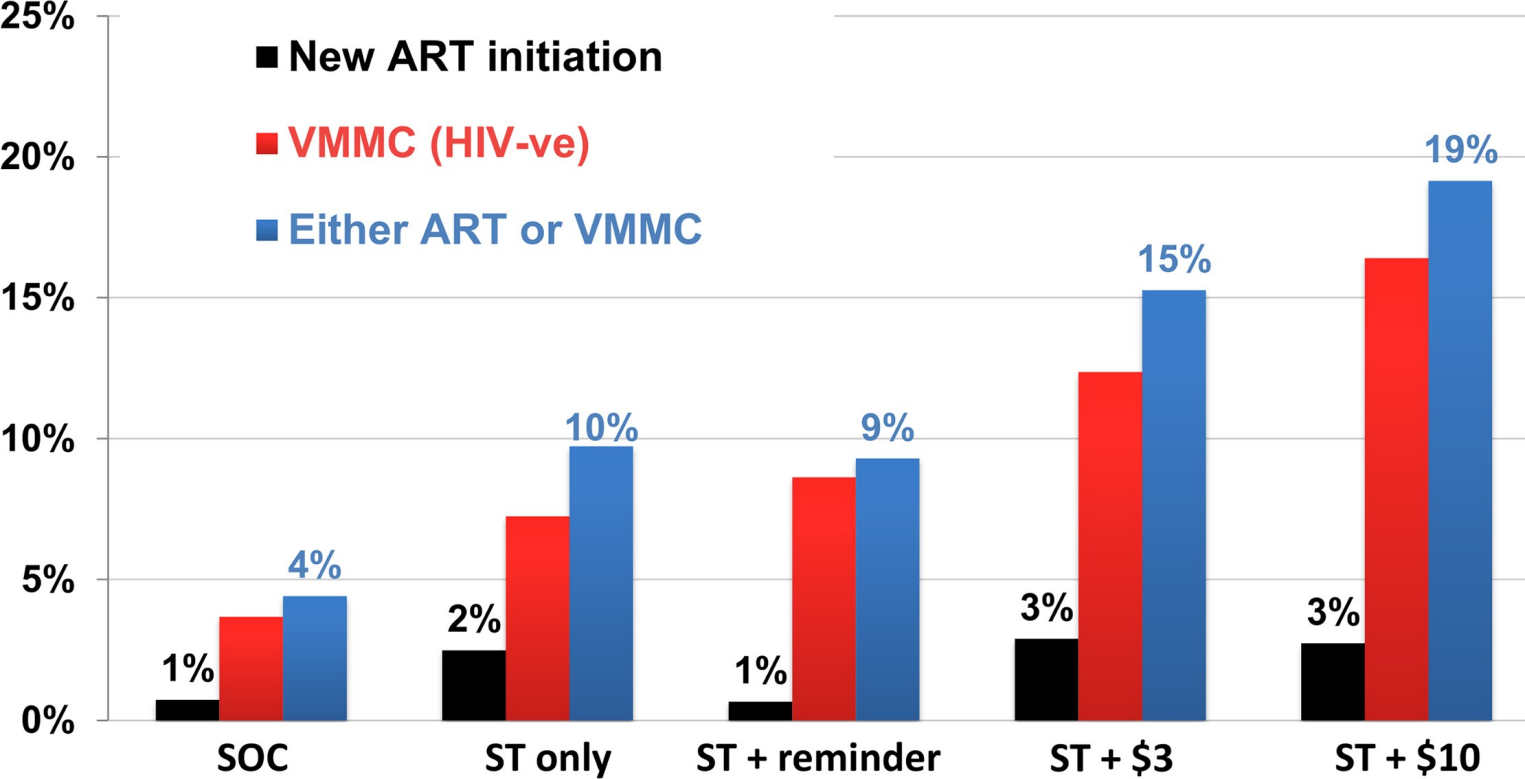
Percentage of all male partners linking to HIV treatment or circumcision (*n* = 2,349 eligible women). Intention-to-treat analysis using the denominator of all eligible women (assumes each woman has 1 male partner). ART, antiretroviral therapy; HIV-ve, HIV negative; SOC, standard of care; ST, self-test; VMMC, voluntary medical male circumcision.

A total of 268 men were either referred for ART as they had tested HIV positive or were referred for VMMC as they had tested HIV negative and were not already circumcised (Table 2). Compared to SOC, the ST + $3 and the ST + $10 interventions were associated with improved referral for ART or VMMC, with aRR 3.06 (95% CI 1.43–6.57) and 3.76 (95% CI 1.76–8.03), respectively. The ST only and the ST + reminder interventions were not associated with significant improvements in this outcome, with aRR 1.41 (95% CI 0.79–2.50) and 1.68 (95% CI 0.90–3.15), respectively. Estimates were similar although larger for the outcome of starting ART or undergoing circumcision (Fig 3).

### Secondary outcomes

In the SOC arm, 71/408 (17.4%) women reported through ACASI that their male partner had had an HIV test following trial enrolment (Table 3). In all intervention arms, partner HIV testing as reported by women was much higher, ranging from 87.0% to 95.4% (*p* < 0.001 for comparison to SOC for all intervention arms).

**Table 3:** Secondary outcomes of male partner HIV testing as reported by women and adverse events within 28 days (stage 1 and 2 combined).

Outcome	Trial arm					
	SOC	ST only	ST + $3	ST + $10	ST + lottery*	ST + reminder
Total women eligible (*n* = 2,349)	408	442	380	512	155	452
Male partner had an HIV test^†^^,^^‡^	71 (17.4%)	407 (92.1%)	345 (90.9%)	483 (94.3%)	135 (87.0%)	431 (95.4%)
Risk ratio (95% CI)	1	5.29 (4.28–6.55)	5.22 (4.21–6.46)	5.42 (4.38–6.70)	5.00 (4.02–6.24)	5.48 (4.43–6.78)
*p*-Value	NA	<0.001	<0.001	<0.001	<0.001	<0.001
Adjusted risk ratio (95% CI)^§^	1	2.75 (1.64–4.63)	4.84 (3.18–7.37)	6.69 (3.46–12.94)	3.56 (2.19–5.79)	4.85 (2.62–8.99)
*p*-Value	NA	<0.001	<0.001	<0.001	<0.001	<0.001
Women experiencing an adverse event^‡^	0 (0.0%)	2 (0.6%)	0 (0.0%)	1 (0.0%)	0 (0.0%)	0 (0.0%)

*Contributed to stage 1 only.

^†^Male partner went to have a test in SOC arm or self-tested in intervention arms, as reported by the woman.

^‡^Reported by women through audio computer-assisted self-interview (ACASI) 4 weeks after enrolment.

^§^Adjusted for male partner history of HIV testing as reported by the woman at the recruitment clinic.

NA, not applicable; SOC, standard of care; ST, self-test.

No SAEs were reported. Three women (2 in ST arm and 1 in ST + $10 arm) reported a grade 2 AE in stage 1, with no AEs reported in stage 2 (Table 3; S3 Appendix). In each case, the AE occurred from women pressurising their partner to use a self-test kit, leading to disagreements culminating in temporary (2–3 days) separation. No physical or sexual violence was reported, and all 3 events resolved within 3 days.

### Cost analysis

In the SOC arm, the average cost per male partner tested for HIV within 28 days was US$9.85, and the average cost per male partner who started ART or underwent VMMC was US$39.81 (Table 4). The average cost per male partner who tested for HIV within 28 days for the 5 intervention arms ranged from US$23.73 (ST + $3) to US$41.24 (ST + reminder). The average cost per male partner who started ART or was referred for VMMC for the 5 intervention arms ranged from US$94.32 (ST + $3) to US$167.95 (ST + lottery).

**Table 4:** Findings from cost analysis.

Resource use item or outcome	Trial arm					
	SOC (*n* = 408)	ST only (*n* = 442)	ST + $3 (*n* = 380)	ST + $10 (*n* = 512)	ST + lottery (*n* = 155)	ST + reminder (*n* = 452)
Antenatal care intervention						
Personnel	$57.52	$67.13	$55.92	$75.07	$22.12	$67.13
Intervention cost*	$0.01	$2,785.98	$2,785.58	$5775.65	$1,007.89	$3,009.29
Total cost	$57.53	$2,853.12	$2,841.50	$5,850.73	$1,030.01	$3,076.42
MFC intervention						
Personnel	$184.74	$273.04	$386.74	$750.29	$66.69	$179.89
Consumables	$226.56	$167.61	$233.26	$445.90	$42.13	$106.59
Equipment	$14.55	$25.02	$35.65	$69.50	$6.05	$16.68
Overhead and other capital^†^	$75.02	$127.24	$181.30	$353.45	$30.77	$84.83
Total cost	$499.87	$592.91	$836.95	$1,619.15	$145.64	$387.99
Total intervention cost^‡^	$557.40	$3,446.03	$3,678.44	$7,469.87	$1,175.64	$3,464.41
Number of male partners who tested for HIV and attended MFC within 28 days^§^	56	85	155	266	30	84
Number of male partners who started ART or were circumcised	14	27	39	68	7	22
Average cost per male partner who tested for HIV and attended MFC within 28 days	$9.95	$40.54	$23.73	$28.08	$39.19	$41.24
Average cost per male partner who started ART or was circumcised^‡^	$39.81	$127.63	$94.32	$109.85	$167.95	$157.47

All costs in 2016 US dollars.

*Depending on trial arm, cost includes information leaflet ± OraQuick HIV Self Test (US$3.23 each) ± financial incentive ± lottery ± phone call reminder.

^†^Includes clinic rental cost, utilities, and refresher training for HIV counsellors.

^‡^Does not include cost of ART or VMMC.

^§^This is the primary outcome of male partner testing and MFC attendance within 28 days.

ART, antiretroviral therapy; MFC, male-friendly clinic; SOC, standard of care; ST, self-test; VMMC, voluntary medical male circumcision.

## Discussion

The main findings of this trial were that, in Blantyre, Malawi, secondary distribution of HIVST kits to male partners by women attending antenatal care greatly increased the proportion of male partners who had an HIV test and, if combined with conditional financial incentives or a phone call reminder, significantly increased male linkage into post-test HIV care and prevention services. With self-testing recently recommended by WHO [3]—and several low-cost products, including 1 WHO prequalified self-test and 4 others approved for procurement through major donors—national strategies based on HIVST such as the one described here are becoming highly feasible.

Pregnancy and the postpartum period are times of unusually high HIV risk for both partners as well as the child, such that reaching the male partners of pregnant women with HIV services has high societal and public health value [11]. In our study, women reported high uptake of HIVST by their male partner at 28 days (range 87.0% to 95.4% in the 5 HIVST intervention arms). Previous ANC-based studies in Kenya [7,17] and Uganda [18], also reliant on proxy-reporting, estimated testing by 90.8% to 91.1% of men using secondary distribution of self-test kits. This is in stark contrast with previously disappointing results for male partner testing initiatives, and is consistent with other data that suggest that HIVST is highly acceptable to men, adolescents, and key populations who are not well served by standard clinic-based HIV testing services [19,20]. HIVST should now be considered as a leading candidate for routine ANC policy and practice in high HIV prevalence settings.

Although merely testing as a couple can reduce sexual risk taking [21], timely uptake of ART, if HIV positive, and effective prevention, if HIV negative, are key to maximising and maintaining the health benefits of all testing modalities [1]. Improving previously poor linkage into HIV care following standard HIV testing has been a major recent focus of HIV programmes globally [22]. For HIVST, reconstructing the standard HIV care cascade [1] is generally not possible [5], but demand for post-test services at the population level (without knowledge of underlying HIV prevalence) provides an alternative measure of effect [6].

Here, using a composite HIV care and prevention outcome, we show significant benefits from 2 conditional fixed financial incentive arms. Financial incentives, including one-off rewards for desired behaviour, have shown a consistently positive effect on uptake and completion of standard HIV testing [23–25]. Interestingly, our results differ in that men’s uptake of HIVST was not influenced by incentives, based on self-report by women. Instead, uniformly high kit usage rates in all self-testing arms suggest high intrinsic motivation for self-testing, to which incentives add relatively little. HIVST has high user acceptability [19], is strongly preferred over alternative approaches [19], and incurs negligible inconvenience or user costs when delivered at home [14]. Combining HIVST with conditional fixed financial incentives did, however, increase the proportion of men attending clinic-based HIV care and prevention services within 28 days, from 13.0% in the SOC arm to 51.7% in the ST + $10 arm. This includes a significant increase in the hard prevention outcomes of ART initiation and VMMC, and adds to the body of mixed evidence concerning incentives and linkage [26–28], as well as establishing the principle that linkage interventions can increase health benefits from secondary distribution HIVST strategies.

Our costing suggests that of the 5 interventions evaluated, the $3 and $10 incentive arms offered the best value for money. Malawians incur a cost of approximately US$3 to access free facility-based HIV testing [14], and this cost often deters men [29]. The $10 incentive in the ST + $10 incentive arm was considered by potential participants as the likely maximally efficacious amount to cover all their opportunity costs [13]. Although providing financial incentives and HIV self-test kits is costly, it may be necessary to reach the UN 2020 targets. The potential to rapidly test and optimise locally relevant financial incentives is a strength of multi-arm adaptive trial designs, modified here to support clustered units of randomisation. For instance, we anticipated but did not find high acceptability/effectiveness of lottery-based incentives following behavioural intervention trials that showed significantly reduced HIV incidence in Lesotho [30] and increased HIV testing in Zimbabwe [31]. Our cost analysis may overestimate the cost per person tested since the number of male partners tested is based on men who attended the clinic while a higher number may have tested without necessarily attending the clinic.

Limitations of the study are the relatively small number of groups of women attending on any single ANC day and randomised to each arm, and proxy-reporting to estimate usage of HIVST by male partners. Computer-based interviews (ACASI), however, were used to minimise misreporting due to social desirability bias [32]. There was no incentive for the SOC participants to attend the MFC except that it was a fast track clinic. This may potentially bias the results towards the incentive arms appearing to be more effective than they truly are as men may otherwise have sought care elsewhere. Risk behaviour, condom use, and retention in care were not evaluated [33], and, due to service availability restrictions, not all men were followed through from booking to VMMC.

In this study we show pronounced effects on testing without safety concerns from secondary distribution of HIVST kits, in keeping with previous estimates from ANC studies, with no additional benefit from accompanying financial incentives. This implies that secondary distribution of HIVST kits through ANCs may be scalable. Incentives of $3 and $10, conditional on attending clinic-based services within 28 days, and a phone call reminder did, however, significantly increase timely uptake of HIV treatment and prevention to an extent likely to be cost-saving. Compared to SOC, we saw no significant benefit of providing HIVST kits only, according to the primary endpoint. Secondary distribution of HIVST kits, ideally accompanied by interventions promoting timely linkage into HIV care and prevention cascades, is a promising new approach for routine ANC services to reach male partners, enhance prevention of mother-to-child transmission, and contribute more broadly to country-level HIV prevention targets.

## Supporting information

S1 CONSORT Checklist Checklist of information to include when reporting a cluster randomised trial. (DOCX)

S1 Appendix Description of statistical analysis plan. (DOCX)

S2 Appendix Cost analysis. (DOCX)

S3 Appendix Adverse events grading table. (DOCX)

S1 Table Adjusted intervention effects for trial arms by trial stage, for the primary outcome. (DOCX)

S1 Text PASTAL MAMS protocol v0.5. (PDF)

S2 Text Statistical analysis plan for PQ40 PASTAL Trial SAP v0.4. (PDF)

## Abbreviations

ACASI: audio computer-assisted self-interview
AE: adverse event
aRR: adjusted risk ratio
ART: antiretroviral therapy
ANC: antenatal care clinic
DSMB: data and safety monitoring board
HIVST: HIV self-testing
MAMS: multi-arm, multi-stage
MFC: male-friendly clinic
RR: risk ratio
SAE: serious adverse event
SOC: standard of care
VMMC: voluntary medical male circumcision
