# Supplementary material for: HIV self-testing alone or with additional interventions, including financial incentives, and linkage to care or prevention among male partners of antenatal care clinic attendees in Malawi: An adaptive multi-arm, multi-stage cluster randomised trial
Source: PLoS Med. 2019 Jan 2;16(1):e1002719. doi: 10.1371/journal.pmed.1002719 (PMC6314606; doi:10.1371/journal.pmed.1002719)
Supplement: S1 Appendix — (DOCX) [file pmed.1002719.s002.docx]

**S1 Appendix:** Description of statistical analysis plan

*Design and participants*

This was an adaptive multi-arm two-stage cluster randomized trial randomising antenatal care (ANC) clinic days (clusters) to one of six trial arms in stage 1. At a pre-planned interim analysis conducted at the end of stage 1, adaptations included sample size re-calculation and dropping intervention arms deemed inefficacious when compared to the standard of care (SOC). New set of clusters with new ANC attendees was randomized to trial arms that proceeded to stage 2. Pregnant women accessing ANC for the first time were given a personalized clinic invitation letter only to give to their male partner (SOC). The self-test (ST) only arm offered SOC with two oral self-test kits only, the ST+$3 and ST+$10 arms offered fixed financial incentives in the local currency equivalent to $3 and $10 to male partners conditional on clinic attendance. The ST+lottery offered a 10% chance to male partners who attended the clinic to win $3 x the number of male partners attended. The final arm was ST+reminder where a phone call reminder was made to the male partner immediately the woman returned from the clinic for the man to use the self-test kits and attend the clinic, repeated after five days. It was assumed that each women had one male partner who she named during enrollment.

*Primary outcome and measurement*

The proportion of male partners of ANC attendees who tested for HIV and attended the clinic within 28 days of enrolling the woman. In SOC, male partners were tested for HIV by an HIV counsellor while in all intervention arms men had to present a used self-test kit or were asked to use it on spot by an HIV counsellor to confirm that HIV testing had occurred. All men with a positive HIV test received additional testing to confirm their results before being referred to a nurse for initiation of HIV treatment. Men who had a negative HIV test result and reported uncircumcised were referred for voluntary medical male circumcision. Already circumcised HIV negative men were counselled. Data collection was done with each trial procedure in order to ascertain the trial outcomes.

*Statistical analysis of primary outcome*

Analysis was by intention-to-treat taking eligible women as the denominator and male partners who achieved the primary outcome as the numerator. Analysis was by cluster-level summaries using the geometric mean of the proportion achieving the primary outcome in each cluster. This was to account for the clustered design given the small number of clusters per arm, but also all analysis adjusted for multiplicity using the Dunnett test. A two-stage analysis approach was used to adjust for covariates that showed imbalance on baseline characteristics: male partner history of HIV testing and recruitment clinic. Firstly, a logistic regression model was fitted with the two covariates to obtain an expected outcome. Secondly, the cluster-level ratio of observed:expected outcomes were compared by arm. A t-test was used to compute a p-value for each comparison with degrees of freedom reduced by 2 to account for the cluster-level variable of recruitment clinic, followed by a Dunnett test to correct for multiple comparisons.

*Deviation from original statistical analysis plan*

The original analysis plan assumed that the two trial stages were independent. Therefore, each stage was analysed separately with estimates and p-values combined. However, the current analysis is based on combined data from both trial stages. This was done because there were no major differences in results between the two analytical approaches and the combined analysis easier to understand for readers.
