# Supplementary material for: HIV self-testing alone or with additional interventions, including financial incentives, and linkage to care or prevention among male partners of antenatal care clinic attendees in Malawi: An adaptive multi-arm, multi-stage cluster randomised trial
Source: PLoS Med. 2019 Jan 2;16(1):e1002719. doi: 10.1371/journal.pmed.1002719 (PMC6314606; doi:10.1371/journal.pmed.1002719)
Supplement: S2 Appendix — (DOCX) [file pmed.1002719.s003.docx]

**S2 Appendix:** Cost analysis

Cost analysis was undertaken from the health provider perspective. For each of the six trial arms we estimated the total cost of providing the intervention to all participants randomised to that arm. This included the costs of providing the intervention at the antenatal clinic and the costs of providing care at the male friendly clinic. We did not include the cost of receiving ART or performing the male circumcision.

For each trial arm we recorded all resources used by those randomised to that arm. We then costed each resource use item to estimate the total costs. The cost of delivering the intervention at the ante-natal clinic included time with an HIV counsellor and invitation letter, and depending on the trial arm, the cost of HIV self-test kits, financial incentives or phone call reminders. As the interventions were delivered to pregnant women during their routine ante-natal clinic visits we did not include the healthcare resources utilized by the pregnant woman. The cost of the male friendly clinic included the cost of: staff salaries; training of staff; consumables and equipment; and overheads. Data from the World Bank were used to adjust all costs to 2016 US Dollars.

We estimated the cost per male partner tested for HIV and attended the male friendly clinic by dividing the total cost of providing the trial intervention to those randomised to that trial arm by the total number of men who tested for HIV and attended the male friendly clinic. We estimated the cost per male partner tested for HIV and either started onto ART or underwent VMMC. We did this by dividing the total cost of providing the trial intervention to those randomised to that trial arm by the total number of men who started ART or referred for VMMC.
