# Supplementary material for: HIV self-testing alone or with additional interventions, including financial incentives, and linkage to care or prevention among male partners of antenatal care clinic attendees in Malawi: An adaptive multi-arm, multi-stage cluster randomised trial
Source: PLoS Med. 2019 Jan 2;16(1):e1002719. doi: 10.1371/journal.pmed.1002719 (PMC6314606; doi:10.1371/journal.pmed.1002719)
Supplement: S3 Appendix — (DOCX) [file pmed.1002719.s004.docx]

**S3 Appendix:** Adverse events grading table

|  | **GRADE 1**  (Mild) | **GRADE 2**  (Moderate) | **GRADE 3**  (Severe)  (Within 30 days) | **GRADE 4**  (Potentially life-threatening)  (Within 30 days) |
| --- | --- | --- | --- | --- |
|  | 1. Verbal, emotional or psychological Intimate-partner violence (IPV) 2. Denying access to household resources 3. Being ignored 4. Being controlled (e.g. not allowed to leave house) | 1. Coercion to self-test. 2. Coercion to disclose a self-test result 3. IPV that includes pushing, or slapping with an open hand that does not result in pain, or visible marks >24hrs 4. Severe or prolonged psychological or emotional IPV leading to disruption of daily activities 5. Psychologically coercive sex | 1. IPV that leads to pain, bruising or marks >24hrs. 2. Threat of life-threatening violence (e.g. statement of intent to kill, mock strangulation, threatened with a knife or gun 3. Physically coercive sex 4. Reports fearing for her life 5. Marriage break-up | 1. IPV leading to hospitalisation or death 2. Suicide or attempted suicide 3. Attack using potentially lethal force (e.g. knife, gun, hammer, kicks to the head) |
| STEPS TO BE FOLLOWED | Refer to community based institutions for assistance. e.g  Church leaders and marriage counsellors | Refer to community-based gender-based violence (GBV) support organisations (One Stop Centre and Queen Elizabeth Central Hospital Counselling Centre) | - Facilitate urgent referral to Queen Elizabeth Central Hospital - Refer to One Stop Centre for psycho-social support - Facilitate relocation to a safe environment | - Facilitate urgent referral to Queen Elizabeth Central Hospital - Ensure safe alternative abode before discharge - Refer to One Stop Centre for psycho-social support - Report to police (Suicide / Murder) |

Grade 1 indicates a mild event -

Grade 2 indicates a moderate event

Grade 3 indicates a severe event

Grade 4 indicates a potentially life-threatening event

Grade 5 indicates death. Not indicated in the table.
