# Supplementary material for: HIV self-testing alone or with additional interventions, including financial incentives, and linkage to care or prevention among male partners of antenatal care clinic attendees in Malawi: An adaptive multi-arm, multi-stage cluster randomised trial
Source: PLoS Med. 2019 Jan 2;16(1):e1002719. doi: 10.1371/journal.pmed.1002719 (PMC6314606; doi:10.1371/journal.pmed.1002719)
Supplement: S1 Table — (DOCX) [file pmed.1002719.s005.docx]

**S1_Table:** Adjusted* intervention effects for trial arm by trial stage for the *primary outcome*

|  | **Trial arm** | | | | | | | | |  |
| --- | --- | --- | --- | --- | --- | --- | --- | --- | --- | --- |
|  | **SOC** | **ST only** | **ST + $3** | **ST + $10** | | **ST + Lottery†** | | **ST + Reminder‡** | |  |
| **First stage** |  |  |  |  | |  | |  | |  |
| Eligible | 198 | 187 | 146 | 216 | | 155 | | 182 | |  |
| Outcome | 27 | 38 | 61 | 102 | | 30 | | 42 | |  |
| Proportion** | 0.124 | 0.179 | 0.428 | 0.473 | | 0.186 | | 0.316 | |  |
| RR | 1 | 1.61 | 3.31 | 3.60 | | 1.53 | | 1.88 | |  |
| 95% CI |  | 0.95; 2.73 | 1.43; 7.66 | 1.34; 9.69 | | 0.93; 2.52 | | 1.13; 3.13 | |  |
| p-value§ | NA | 0.211 | 0.001 | 0.001 | | 0.211 | | 0.039 | |  |
| **Second stage** | | | | | | | | | |  |
| Eligible | 210 | 255 | 234 | 296 | | Dropped | | 270 | |  |
| Outcome | 29 | 47 | 94 | 164 | |  | | 42 | |  |
| Proportion** | 0.135 | 0.172 | 0.394 | 0.558 | |  | | 0.165 | |  |
| RR | 1 | 1.13 | 2.61 | 3.83 | |  | | 1.51 | |  |
| 95% CI |  | 0.67; 1.92 | 1.13; 6.04 | 1.42; 10.31 | |  | | 0.91; 2.49 | |  |
| p-value§ | NA | 0.629 | <0.001 | <0.001 | |  | | 0.148 | |  |
| * Adjusted for male partner past HIV testing history as reported by the woman and recruitment clinic | | | | | | | | | |  |
| *Primary outcome:* of evidence of testing and male friendly clinic attendance within 28 days (regardless of HIV test result) | | | | | | | | | |  |
| SOC: standard of care; ST: self-test; RR: risk ratio | | | | | |  | |  | |  |
| † 10% chance of winning $3 times number of men achieving the primary outcome | | | | | | | | | |  |
| ‡ phone call |  |  |  |  | |  | |  | |  |
| § Adjusted for multiple comparisons using the Dunnett’s test | | | | |  | |  | |  | |
| ** Geometric mean of the cluster proportions | | | | | |  | |  | |  |
