# Supplementary material for: HIV self-testing alone or with additional interventions, including financial incentives, and linkage to care or prevention among male partners of antenatal care clinic attendees in Malawi: An adaptive multi-arm, multi-stage cluster randomised trial
Source: PLoS Med. 2019 Jan 2;16(1):e1002719. doi: 10.1371/journal.pmed.1002719 (PMC6314606; doi:10.1371/journal.pmed.1002719)
Supplement: S1 Text — (PDF) [file pmed.1002719.s006.pdf]

Investigating interventions to increase uptake of HIV testing and linkage into care or prevention for male partners of pregnant women in antenatal clinics in Blantyre, Malawi: an adaptive Phase II multi-arm multi-stage cluster randomised trial

Short title: Partner-provided self-testing and linkage (PASTAL)

Trial registry: International Standard Randomised Controlled Trial Number (ISRCTN)

Funder: Wellcome Trust, UK

Principal Investigator: Augustine Choko (Wellcome Trust Fellow in Public Health and Tropical Medicine)

Host Institute: Malawi-Liverpool-Wellcome Trust Clinical Research Programme (MLW)

Training Institute: London School of Hygiene & Tropical Medicine (LSHTM)

Local collaborators: -

Malawi Liverpool Wellcome Trust Clinical Research Programme (MLW) and College of Medicine

Prof Elizabeth Corbett: Epidemiological input and Sponsor

Dr Nicola Desmond: Social Science input

Mr Moses Kumwenda: Social Science input

Ministry of Health (MoH) Collaborators

Department of HIV/AIDS: Mr Simon Makombe, MoH input

Blantyre District Health Office: Medson Matchaya, local MoH involvement

International Collaborators: -

Dr Katherine Fielding; Dr Aurelia Lepine **LSHTM, UK**
Statistical input; Health Economics input

Prof Nigel Stallard; Dr Hendramoothy Maheswaran **WMS, UK**
Statistical input (Adaptive Designs); Health Economics input

Version 0.5 last amended 03/06/2016 by ATC

Signature: Date: 03/06/2016

Institutions under whose umbrella the research project will be conducted:

Malawi Liverpool Wellcome Trust Clinical Research Programme (MLW)

London School of Hygiene and Tropical Medicine (LSHTM)

Liverpool School of Tropical Medicine (LSTM)

Warwick Medical School (WMS)

Table of Contents

1. Executive Summary 6
2. Background 7
3. Study Rationale 8
4. Research Question, Objectives and Study Design 8
   4.1 Research Question 8
   4.2 Objectives 8
5. Methods 9
   5.1. Study Design and Setting 9
   5.2. Study Population 9
   5.3. Randomisation, Concealment and Blinding 10
   5.3.1. Randomisation 10
   5.3.2. Concealment 11
   5.3.3. Blinding 11
   5.4. Primary Outcome 11
   5.5. Secondary Outcomes 11
   5.6. Trial Procedures 11
   5.6.1. Standard of Care arm 11
   5.6.2. Intervention arms 11
   5.7. Outcome measurement 12
   5.8. Sample Size Considerations 14
   5.8.1. Overall study power 16
6. Study Period (Timelines) 17
7. Data Management and Statistical Analysis 18
   7.1. Data Management 18
   7.2. Statistical Analysis 18
   7.2.1. Stage 1 Statistical Analysis 19
   7.2.2. Stage 2 Statistical Analysis 19
8. Data Safety Monitoring Board (DSMB) 20
   8.1. DSMB 20
9. Ethics, Conflict of Interest, Data Availability and Dissemination 21
   9.1. Ethical Approval 21
   9.2. Conflict of interest 21
   9.3. Data Availability 21

9.4. Dissemination ... 21
10. Possible Constraints ... 22
11. Training and Capacity Building ... 22
12. Personnel, Materials and Consumables, Equipment, Space and Miscellaneous ... 23
12.1. Personnel ... 23
12.2. Materials and Consumables ... 23
12.3. Equipment ... 23
12.4. Space ... 23
12.5. Miscellaneous ... 23
13. Budgetary Estimate ... 24
14. References ... 25
15. Appendices ... 29
A. Investigators' Short CVs ... 29
B. PQ05: Baseline Questionnaire for Women ... 30
C. PQ06: Follow-up Questionnaire for Women ... 33
D. PQ07: Questionnaire for Men who Link ... 36
E. PQ09: Generic Costing Questionnaire ... 39
F. PQ22a: Information Sheet and Consent Women (SOC) -English ... 40
G. PQ22b: Information Sheet and Consent Women (SOC) – Chichewa ... 40
H. PQ23a: Information Sheet and Consent Women (Self-Test Kits only Arm) -English ... 40
I. PQ23b: Information Sheet and Consent Women (Self-Test Kits only Arm) – Chichewa ... 40
J. PQ24a: Information Sheet and Consent Women (Self-Test Kits + a Low Financial Incentive Arm) -English ... 40
K. PQ24b: Information Sheet and Consent Women (Self-Test Kits + a Low Financial Incentive Arm) – Chichewa ... 40
L. PQ25a: Information Sheet and Consent Women (Self-Test Kits + a High Financial Incentive Arm)-English ... 40
M. PQ25b: Information Sheet and Consent Women (Self-Test Kits + a High Financial Incentive Arm) – Chichewa ... 40
N. PQ26a: Information Sheet and Consent Women (Self-Test Kits + a Lottery Financial Incentive Arm) -English ... 40
O. PQ26b: Information Sheet and Consent Women (Self-Test Kits + a Lottery Financial Incentive Arm) – Chichewa ... 40
P. PQ27a: Information Sheet and Consent Women (Self-Test Kits + a Phone Reminder Arm) - English ... 40

Q. PQ27b: Information Sheet and Consent Women (Self-Test Kits + Phone Reminder Arm) – Chichewa 40
R. PQ41: Data Management Plan (DMP) 41
S. PQ42: Data Safety Monitoring Board (DSMB) Charter 46
T. Blantyre DHO Letter of Support 57

1. Executive Summary

Background: Wide roll out of availability of HIV tests has led to increases in numbers testing and starting on antiretroviral treatment (ART) in sub-Saharan Africa. Despite such remarkable progress, men continue to lag behind in HIV testing in the region including men in well-established relationships where HIV transmission is surprisingly high. It is therefore important to investigate interventions to improve both testing and linkage into care or prevention for men. We found HIV self-testing (HIVST) very effective at increasing the uptake of testing in the general population in Blantyre, Malawi. Here we propose a study based on HIVST delivered through the woman, to her male partner, to investigate additional interventions aimed at increasing both the uptake of testing and linkage into care or prevention for male partners of pregnant women attending antenatal clinics (ANC) in Blantyre, Malawi.

Aim: To investigate multiple candidate interventions based on HIV self-testing in a Phase II trial in order to select ≤ 3 interventions for inclusion in a potential Phase III study.

Participants: Pregnant women attending ANC at Ndirande, Zingwangwa and Bangwe primary health centres as well as their male partners.

Design: A Phase II adaptive multi arm multi stage (MAMS) cluster randomised trial (CRT) using ANC clinic day as the unit of randomisation. The trial will have one interim analysis followed by final analysis (2-stage MAMS design). The first stage will have at most six arms: standard of care (SOC) and five intervention arms; HIV self-test kits (ST) only, ST + a fixed financial incentive equivalent to transport cost ($3), ST + a higher financial incentive ($10), ST + a lottery-based financial incentive, ST + phone call reminder.

**Primary outcome:**	Proportion of male partners who test for HIV and link into care or prevention within 28 days.
**Secondary outcomes:**	Proportion of women who participate in their allocated study arm.
	Proportion of women who self-report delivering STs to their partners.
	Proportion of male partners who test for HIV within 28 days.
	Cumulative incidence of intimate partner violence associated with each study arm.
	Total cost of implementing the service per study arm.

Six clinic days per arm (total of 36) will be required for the first stage assuming a difference in means of 0.15 in the primary outcome between SOC and an intervention arm with a coefficient of variation (k) of 0.10. At the end of the first stage, intervention arms that are not significantly better than the SOC will be dropped. Sample size for the second stage will be re-calculated based on empirical estimates from the first stage. A t-test will be applied to the mean of clinic day proportion estimates for each intervention arm compared to the SOC for the primary outcome. The Dunnett test and the weighted inverse normal method will be used to control for multiple-comparisons and multiple stages, respectively.

Consent and confidentiality

Written informed consent, or witness consent plus thumb-print if illiterate, will be taken from all women. Waiver of written consent will be sought from the institutional review boards for the male partners.

Intended use of results and dissemination

The results will help us understand which interventions hold potential to increase the uptake and linkage into care or prevention for male partners of pregnant women. Results will be disseminated to HIV Unit in Malawi Ministry of Health, College of Medicine in Blantyre, and through conference presentations and publication in peer-reviewed journal.

2. Background

Sub-Saharan Africa (SSA) accounts for 70% of the global HIV burden despite rapid scale up of HIV services including testing[1]. However, analysis of the HIV care cascade indicates a striking falloff in numbers between testing and linkage into care or HIV prevention[2-4]. Men regularly feature among populations with lower uptake of HIV testing across SSA[5] and lower rates of linkage into care or prevention[6] in the era of extremely ambitious targets for HIV[7]. The situation is worse among male partners of antenatal clinic (ANC) women attendees, with less than 35% undergoing HIV testing when invited through their partner[8-10]. African women face substantial risk of new infection (3.6% per pregnancy in study cohorts) that carries unusually high risk to the infant[11]. Pregnancy *per se* doubles the risk of transmitting HIV to an uninfected partner[12] but also presents an opportunity to reach both partners with HIV testing and counselling (HTC) services[13].

A number of different strategies for improving the uptake of HIV testing among male partners of ANC attendees have shown effect: these include home-based testing[14, 15], provider initiated testing and counselling (PITC)[16], couples testing during antenatal visits[17] and home-based couple or partner testing[9, 18]. Key limitations of these strategies include: logistical difficulties of wide scale implementation where home visits are required, lack of convenience, costs, lack of confidentiality and failure to prioritise men's own health[19-21]. HIV self-testing (HIVST) is an alternative approach with potential to increase couple or partner testing[22] and was found to be highly acceptable to men in Malawi[23, 24].

We define *HIVST-plus* as offering HIV self-testing along with an additional intervention aimed at improving linkage into care or prevention. Such additional interventions include facilitated linkage[25], financial incentives (FI)[26-28], and short messaging services (SMS)[29-31]. The wider increase in male engagement may then lead to increased utilisation of prevention-of-mother-to-child transmission (PMTCT) programmes in Africa[8, 9] and improve maternal outcomes[32].

A myriad of candidate interventions exist for the intervention part of HIVST-plus which could maximise both uptake and linkage into HIV care or prevention. For example, different levels or forms (fixed or lottery) of financial incentives [26, 27, 33]. This wide range of interventions presents technical challenges related to appropriate study design and analysis methods in order to identify optimal strategies[34]. Such complexity can be handled by applying adaptive trial designs, which are more flexible (allow pre-specified adaptations at interim analysis) as well as more efficient (time and cost) than standard parallel designs[35, 36]. In multi-arm multi-stage (MAMS) designs, several interventions are compared to a control arm using interim analysis[35], providing an unbiased approach to investigating and selecting multiple Phase II candidates under consideration for Phase III trial[36]. Although predominantly used in the pharmaceutical industry to date, adaptive trial designs could have value in public health evaluations.

We aim to conduct a Phase II adaptive MAMS cluster randomised trial (CRT) randomising clinic days (not individual women) to identify leading HIVST-plus candidate interventions for male partners of ANC attendees in Blantyre, Malawi. To our knowledge, this is the first study to focus on linkage into care or prevention as the main outcome although a number of other studies are planned (Kahn K; South Africa) or underway[37] investigating the uptake of HIVST for male partners of pregnant women.

3. Study Rationale

HIV transmission in Africa remains unusually high in stable sexual relationships with low HIV risk perception, including in married couples[38]. Uptake of HIV testing among male partners of pregnant women remains unacceptably low[10], despite concerted efforts to improve the situation. Therefore, novel approaches should be investigated, such as the ones proposed here.

4. Research Question, Objectives and Study Design

4.1 Research Question

What are the most promising candidate interventions for increasing uptake of HIV testing and linkage into care or prevention for partners of pregnant women attending antenatal clinics in Blantyre, Malawi?

4.2 Objectives

Primary objective

1) To identify the most promising interventions for increasing both the uptake of HIV testing and linkage into HIV care or prevention **among male partners of pregnant women attending ANC**.

This is the only objective that will be examined at interim analysis.

Secondary objectives

2) To identify the most promising interventions for increasing the uptake of HIV testing among **male partners of pregnant women attending ANC.**
3) To assess the acceptability of partner-provided HIVST-plus, as defined by willingness to deliver HIVST kits to male partners **among women attending ANC.**
4) To investigate the risk of serious adverse events within 30 days of enrolment **among women attending ANC and their male partners** who participate in the study.
5) To provide the cost associated with implementation of the service for **each study arm**

5. Methods

5.1. Study Design and Setting

A Phase II adaptive MAMS CRT using ANC clinic day as the unit of randomisation. The trial will have one interim analysis followed by final analysis (2-stage MAMS design). The first stage will have at most six arms: one standard of care (SOC) arm and five intervention arms (Fig. 2). At the end of the first stage, intervention arms that are not significantly better than the SOC will be dropped. Alternatively, intervention arms may also be dropped due to incidence of adverse events (intimate partner violence).

Fig. 1: Schema of the Phase II adaptive MAMS CRT

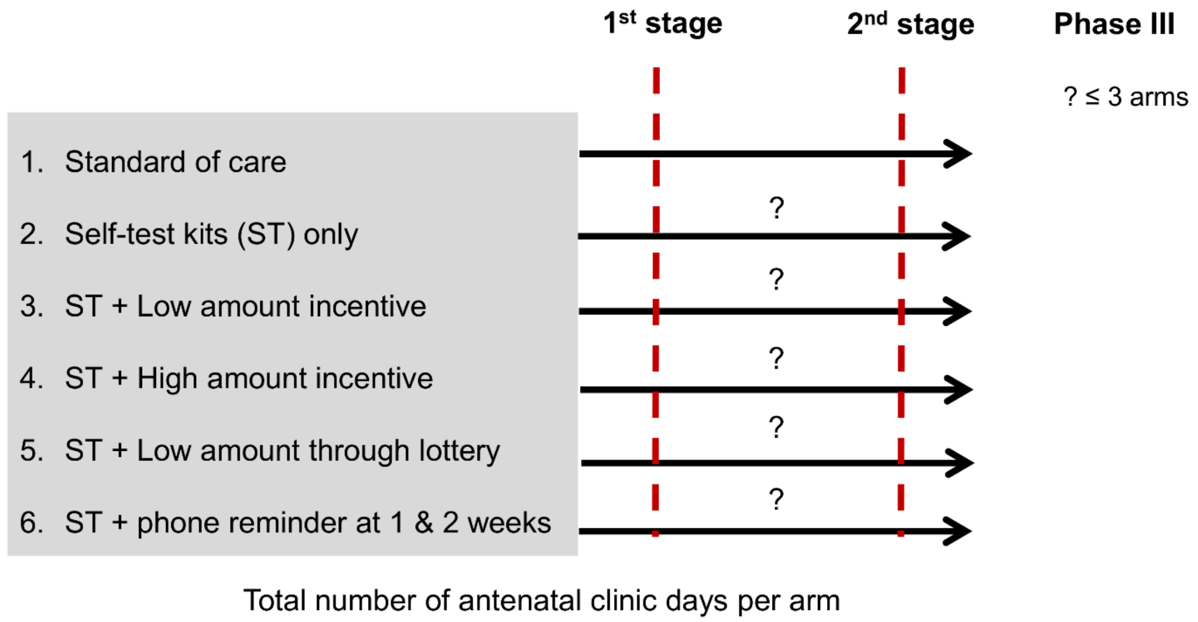

5.2. Study Population

The study will recruit participants from Ndirande, Zingwangwa and Bangwe primary health clinics (PHC) in urban Blantyre, Malawi.

Inclusion criteria

All women attending antenatal clinic for the first time at Ndirande PHC in urban Blantyre and their male partners.

Exclusion criteria

i) Have had couple or partner testing in this pregnancy.
ii) <18 years old.
iii) The man is already aware of their HIV positive status and receiving treatment.
iv) Subsequent ANC visit
v) Already recruited in this trial.
vi) Not urban Blantyre resident.

5.3. Randomisation, Concealment and Blinding

5.3.1. Randomisation

Using clinic day as the unit of randomisation (Fig. 2), each arm will recruit participants from ANC using an allocation sequence. An independent statistician (Dr Mavuto Mukaka) will use computer-generated random numbers to generate the allocation sequence to the study arms. The R package blockrand[39] will be used to create block randomisations of the clinic days across the study arms. The file containing the randomisation sequence will only be accessible to the principal investigator and the independent statistician.

Field workers will enrol participants into each of the six study arms on each clinic day after getting the randomisation allocation for that clinic day. Women will be assessed for eligibility before undergoing group counselling and explanation of the study procedures for that clinic day. All women who show interest at this stage will provide verbal consent to participate followed by written or witnessed thumb print consent. In all five intervention arms, the woman will receive self-test instructions and two self-test kits to take “home”. The test kits, test instructions and a letter will be delivered to the male partner by the woman in order to initiate dialogue for him to test and link into care or prevention as appropriate.

Fig. 2: Randomisation, recruitment, follow-up and outcome evaluation

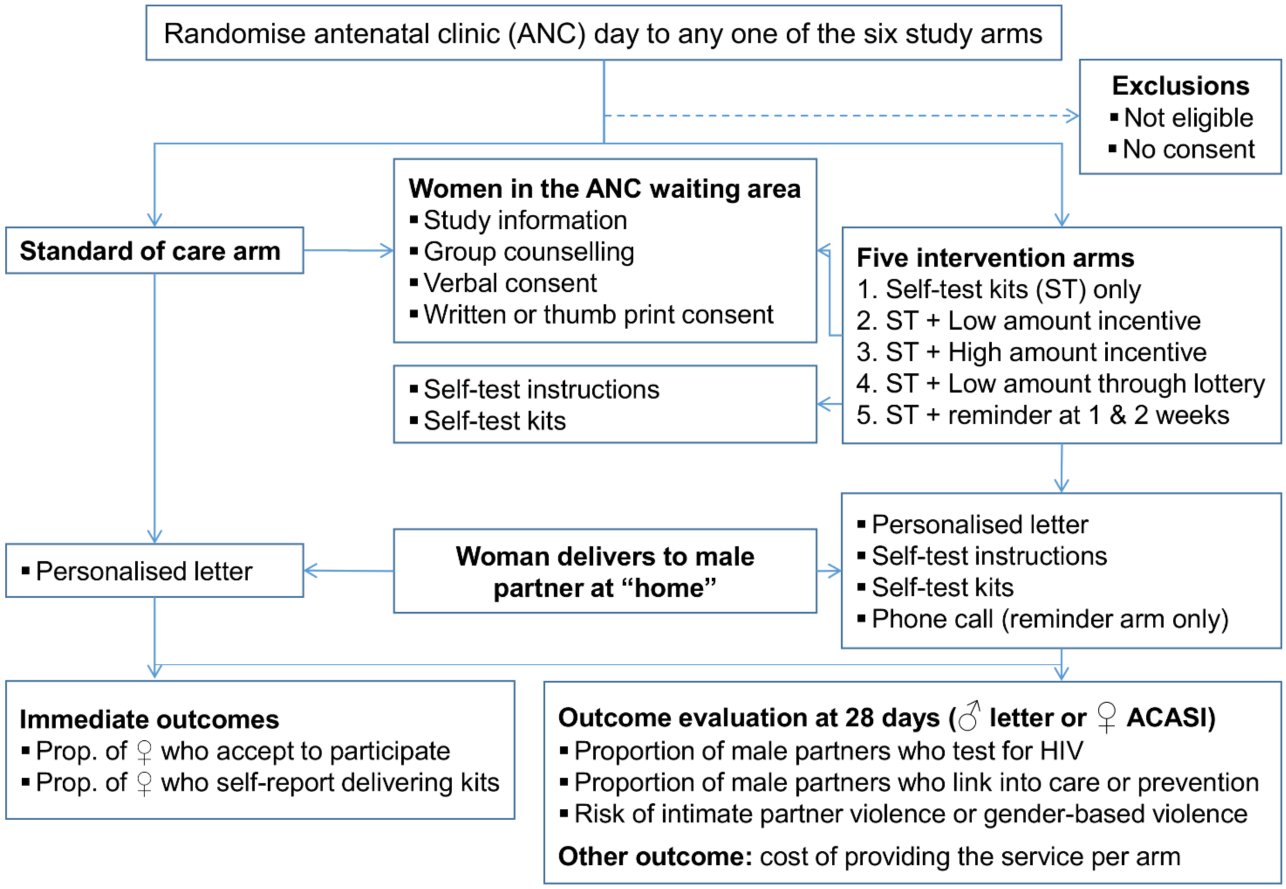

ACASI: audio computer assisted self-interview

5.3.2. Concealment

The randomisation sequence for each clinic day will be provided by telephone on the morning of each clinic day to ensure that study personnel responsible for enrolment do not predict the next arm to recruit.

5.3.3. Blinding

It is not practical to blind either the participants or the investigator in this study because of the nature of the interventions which involve behavioural aspects such as financial incentives. However, the investigators will be blinded to the proportion with the primary outcome per arm such that they will only know the total number of events in the trial and not stratified by trial arm.

5.4. Primary Outcome

1) Proportion of male partners of ANC attendees who test for HIV and link into care or prevention within 28 days. See 5.7 for full description.

5.5. Secondary Outcomes

2) Proportion of male partners of ANC attendees who test for HIV within 28 days.
3) Proportion of women who accept to participate in their allocated study arm.
4) Risk of serious adverse events within 30 days of enrollmentassociated with each study arm.
5) Total cost of implementing the service per study arm.

5.6. Trial Procedures

5.6.1. Standard of Care arm

Women in the SOC arm will receive personalised letters to their male partners only (Fig. 1).

5.6.2. Intervention arms

Besides the personalised letter women in all the five intervention arms will receive at least 2 self-test kits (depending on the number of male partners reported) (Fig. 2).

Women in the self-test kits (ST) only arm will get a personalised letter and self-test kits to deliver to their male partner to encourage him to test and link into MFC. In the reminder arm, the woman will deliver the self-test kits and the personalised letter, and the male partner will get a phone call at one week and at two weeks to remind him to test and link into the MFC. The phone number for the study team will be written on the letter to the male partner in order to enable those without a cell phone to contact the team by sending a free “please call me” message. Up to 90% of people in this setting reported having a working cell phone (unpublished data from a 2015 survey from the same setting). Women who refuse to give the cell phone number for their male partner will be deemed to have refused participation.

In the two fixed financial incentive (FI) arms, the woman will get a personalised letter and self-test kits to deliver to her partner. Should the partner self-test (test) and link into the MFC, he will receive an equivalent of $3 or $10 if he is in the low and high FI arm, respectively. Male partners who link into care or prevention in the lottery FI arm will have a 10% probability of winning an amount equivalent to the low amount arm. Every 1 in 10 male partner who meets the conditionality for the primary outcome (Section 5.7) will get $30. All financial incentives will be disbursed as cash through

mobile money in the trial, with mobile money (money transfer facility operated by mobile phone network providers) chosen for the safety of the staff.

5.7. Outcome measurement

**Primary outcome definition**
A male partner with evidence of HIV testing within 28 days [either presenting with a used self-test kit or undergoing on spot HIV testing with a study HIV counsellor] AND being in pre-ART, received ART, received condoms, or undergone voluntary male medical circumcision (VMMC).

Regardless of trial arm, all male partners will present a letter to the HTC counsellor running the male friendly clinic (MFC). A costing tool validated in urban Blantyre[40] will be used to capture the costs associated with providing the service in each trial arm. The risk of intimate partner violence and proxy-reported male partner HIV testing will be measured through audio computer assisted self-interview (ACASI) administered with the woman at her next (28 days) ANC visit. The primary outcome of testing and linkage into care or prevention will be objectively measured as detailed below.

For all male partners self-reporting an ***HIV positive*** result:

- The HTC counsellor will re-read a used self-test kit if the participant returns one as evidence of self-testing in arms providing HIV self-test kits.
- The HTC counsellor will conduct confirmatory HIV testing in parallel using Determine 1/2™ and Uni-Gold® as well as post-test counselling and providing condoms.
- A nurse will assess all confirmed HIV positive participants for ART eligibility including WHO staging and point of care CD4 testing using Alere Pima™. Participants who are eligible (CD4 <500 or WHO stage 3/4) will be initiated on ART same day while those who are not eligible will be registered for pre-ART. The trial will follow test-and-treat i.e. initiate ART for all participants with a confirmed HIV positive result regardless of CD4 and WHO stage as soon as this policy is effected by the Ministry.

For all male partners self-reporting an ***HIV negative*** result:

- The HTC counsellor will re-read a used self-test kit if the participant returns one as evidence of self-testing in arms providing HIV self-test kits.
- The HTC counsellor will offer testing in all arms using the national serial algorithm with Determine 1/2™. Testing will be waived for participants who return used self-test kit (s) as evidence of self-testing in arms providing HIV self-test kits.
- The HTC counsellor will provide post-test counselling and give condoms.
- The HTC counsellor will link uncircumcised men free voluntary male medical circumcision provided through Population Services Internal (PSI) or the clinic.

For all male partners who link into the MFC in the ***standard of care arm or without having already (self)-tested***:

- The HTC counsellor will offer and conduct HIV testing and counselling using the national serial algorithm with Determine.

All financial incentives are ***conditional*** on initiating ART or being in pre-ART as reported by an ART nurse and reflected in their respective register for participants with an HIV positive test, and on the male partner receiving counselling, condoms or VMMC as detailed above.

Serious adverse events

All adverse events temporally related to participation in each trial arm particularly following delivery of HIV elf-test kits to the male partner will be captured according to a standard operating procedure for handling adverse events (PQ55). The trial will only use the *grading* of adverse events: grade 1 (*mild), grade 2 (moderate), grade 3 (severe),* and *grade 4 (potentially life threatening) or grade 5 (death) in* order to classify all adverse events. Although all adverse events will be recorded only grade 3-5 events, considered as serious adverse events (SAEs) will be reported to the Data Safety and Monitoring Board (DSMB) as defined below:

**Definition of serious adverse event**
Any *grade 3, 4* or *5* events that occur within 30 days of delivering self-test kits to women or men enrolled in the trial.<br><br>**Grade 3 events:**<br>1. Intimate partner violence that leads to pain, bruising or marks within 24hrs.<br>2. Life-threatening violence (e.g. statement of intent to kill, strangulation, threatened with a knife or gun.<br>3. Physically coercive sex.<br>4. Reports fearing for her life.<br>5. Marriage break-up.<br><br>**Grade 4 events:**<br>1. Intimate partner violence leading to hospitalisation or death.<br>2. Suicide or attempted suicide.<br>3. Attack using potentially lethal force (e.g. knife, gun, hammer, and kicks to the head).<br><br>**Grade 5:**<br>1. **Death**

5.8. Sample Size Considerations

A modification of the formula for sample size calculation for a MAMS design for binary outcomes[41] was made based on the methodology for CRTs[42] to identify each stage of the trial. For a 2-sided hypothesis test as will be the case in this trial**,** the formula below calculates the required number of clusters per arm.

$$C_i^c = 1 + \frac{\left(Z\alpha_{i/2} + Z_{\omega_i}\right)^2 \left[\frac{\pi_i^c(1-\pi_i^c)}{m} + \frac{\pi_i^1(1-\pi_i^1)}{m} + k_i^2(\pi_i^c + \pi_i^1)\right]}{(\pi_i^1 - \pi_i^c)^2}$$

C_i^c is the number of clusters in the control arm at $i = 1, 2, \ldots, n$ interim analysis.

$\propto_i$ is the significance level in stage $i = 1, 2, \ldots, n$

ω_i is the type II error (β) rate in stage $i = 1, 2, \ldots, n$.

m is the number of participants per cluster (assumed to be equal in all clusters).

π_i^c is the true proportion of participants with the outcome in the control arm in stage $i = 1, 2, \ldots, n$.

π_i^1 is the true proportion of participants with the outcome in the intervention arm in stage $i = 1, 2, \ldots, n$.

k_i is the between cluster coefficient of variation per stage (assumed to be equal in both the control and the intervention arms per stage).

Six clinic days (total of 36 for 6 arms) per arm would be needed to detect an absolute difference of 15% in linkage compared to linkage in SOC arm of 25% at multiple-comparison adjusted significance level of 0.11 with 80% power in the first stage assuming a coefficient of variation (k) of 0.10[25] (Table 1).

Sample size for the second stage will be re-calculated based on empirical estimates at interim analysis with significance level of 0.1 and 80% power. As a guide, 8 clinic days per arm (total of 32) will be needed assuming that at most 3 arms including the SOC proceed into stage 2.

We expect very little clustering between ANC days but a potential source of variation between clusters will be the different socio-economic and demographic characteristics of the respective catchment populations of the three primary health clinics. Again little variation is expected between clinics as the underlying populations are all poor.

We have made no adjustment for multiple comparisons in calculating sample size as this would lead to unnecessarily large sample size[41]. A larger than conventional (0.05) FWER of 0.2 and 0.1 for stage 1 and stage 2, respectively may lead to erroneously taking forward an ineffective intervention. Being a Phase II trial this is not a major concern as it guards against dropping interventions that may otherwise prove to be effective in a larger, Phase III trial. However, a more conventional type II

FWER of 0.2 was chosen to ensure that there is a high chance of taking forward most of the efficacious interventions from stage 1.

Table 1: Sample size assumptions and parameters

**Assumption**	**1st stage**	**2nd stage**
Linkage into care or prevention in SOC arm by 28 days is 25%	Yes	Yes§
Coefficient of variation (k)	0.10	0.10§
Range for number of women per clinic day	40-60	40-60
% Both woman and man eligible	90%	90%§
% of women who accept to participate per arm	60-90%	60-90%§
Number of male partners per clinic day†	20	20§
**Parameters**		
Family-wise error rate (α)‡	0.20	0.10
Power (1-β)	0.80	0.80§
Allocation ratio (SOC : intervention)	1:1:1:1:1:1	1:1:1§

† Calculated with worst case scenario: 40 women present per clinic day x 0.9 x 0.6
‡ Higher values chosen to get smaller sample size being a Phase II trial
§ May change at interim

5.8.1. Overall study power

Simulation of 1,000 two-stage Phase II trials was set up to assess the probability of finding at least one intervention that is more efficacious compared to SOC at the end of stage two (aka *minimal power),* and the proportion of times intervention arms 2-6 were dropped at interim analysis. A high overall study power is recommended for MAMS trials in order to guard against false negative results[43]. Three intervention arms whose absolute difference in means with the SOC arm was less than 0.06 were most likely to be dropped with 22-44% chance of not proceeding to stage 2. The minimal power when assessed using the stage 2 $\alpha = 0.1$ was 98.4% (Fig 3). Under the trial assumptions and the operating characteristics this trial will be able to identify at least one intervention that can improve linkage into care or prevention that can be tested in a potential Phase III trial.

Fig. 4: Minimal study power at final analysis

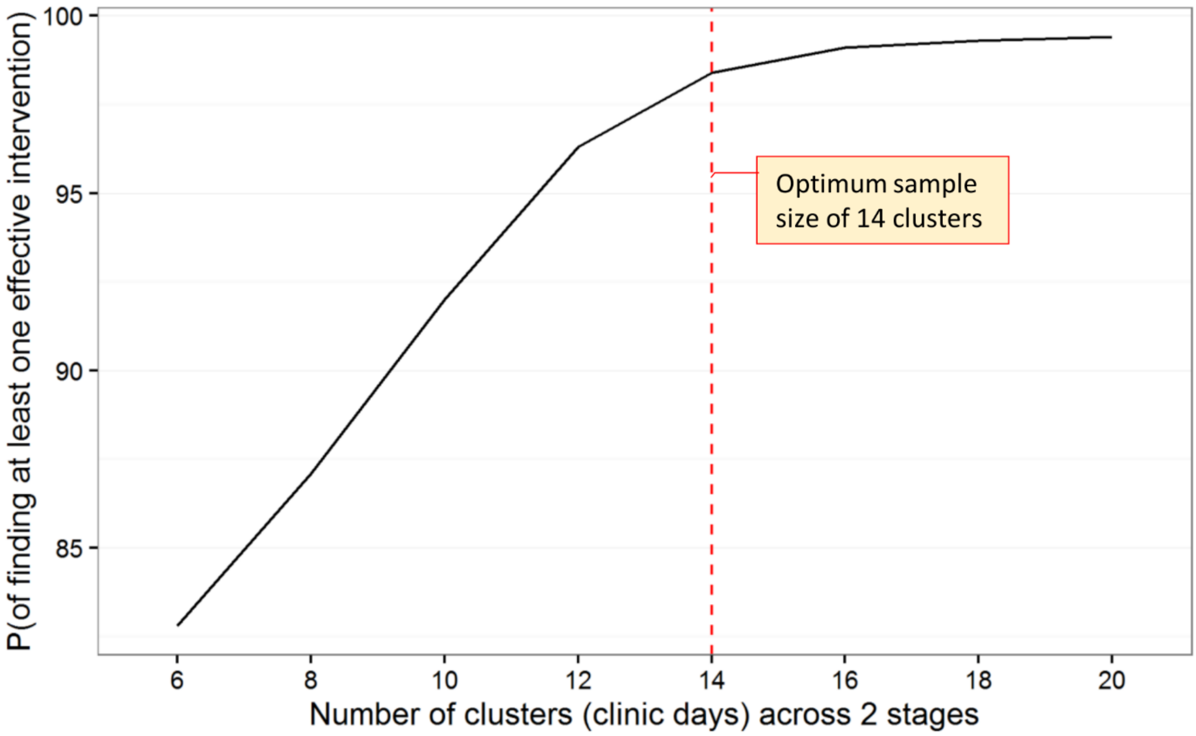

- Vertical line at 16 clusters represents the optimum sample size with family-wise type II error of 0.2 and difference in means of 0.15 between standard of care and most efficacious intervention.
- Allows for multiple comparisons with the standard of care arm

6. Study Period (Timelines)

This trial is planned to run for a total of 12 months (Fig. 4). We plan to seek ethics approval in March 2016 and commence data collection in July 2016 with a Data Safety and Monitoring Board (DSMB) meeting in August 2016 to make key decisions about whether or not to drop arms. Data collection for the 2nd stage is planned to start in September 2016 followed by final data analysis and write up in February 2017. Each stage is planned to recruit and complete outcome measurement within 3 months.

Fig. 4: Timelines

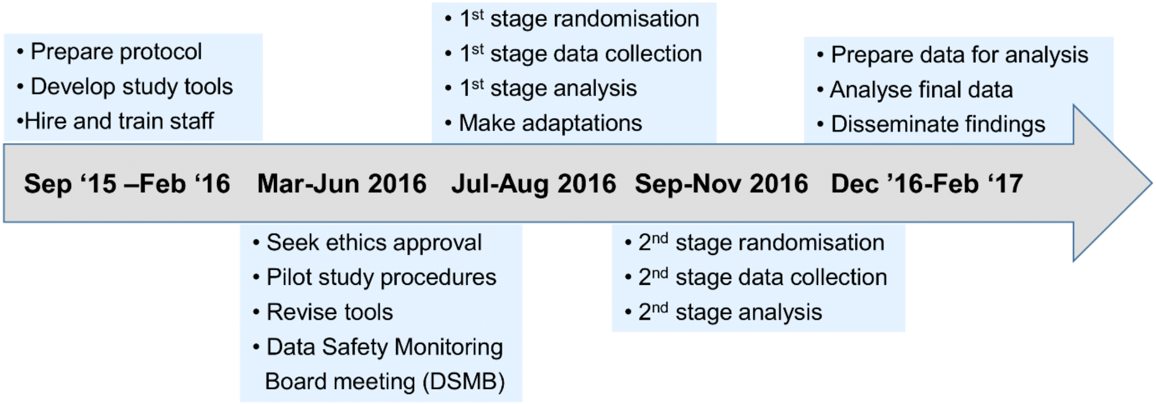

7. Data Management and Statistical Analysis

7.1. Data Management

Data will be managed through infrastructure set up within Malawi-Liverpool-Wellcome Trust Clinical Research Programme (MLW). Data collection and processing will be as detailed in the data management plan (DMP) included in Appendix R. Data will be collected using tablets running Open Data Kit (ODK) and will be downloaded onto a server running a MySQL Relational Database. Data quality assurance will be implemented within the electronic form so that out of range values, inconsistent values and required variables will be checked at the time of data collection. All tablets will have full log-in details of the person collecting the data including a password. Access to the study database will be protected by a password known only to the PI (Augustine Choko) and the IT systems administrator in MLW. Data for study monitoring will be periodically exported into comma separated values (CSV) from the study database on the MLW server for analysis and to raise plus resolve data queries.

Protocols for managing data without breach of confidentiality are in place within MLW. Access to the final data set will be limited to the PI (Augustine Choko), co-principal investigators and the Data Safety and Management Board (DSMB). Sensitive information (including HIV results) will not be linked to personal identifiers in the final data set. All devices and paper-based tools containing data will be kept in locked offices at MLW during data processing and in a locked data repository room for longer term storage. All data will be backed up daily by the MLW Data Office, with offsite back up once weekly. Backup data will be stored in a locked filing cabinet away from the office by the PI.

Data capture tools

These are shown in Appendices B-E, although the final version may have system generated fields from the tablet that are not included. At enrolment a baseline ANC questionnaire will be administered, including eligibility screen. At 28 days a questionnaire will be administered with the women through ACASI aiming to capture adverse events (intimate partner violence) and testing by the male partner. All men who link into care or prevention will be asked to complete a standardised questionnaire including views on the HIVST process and linked services.

7.2. Statistical Analysis

Analyses will be done in R[44] and Stata 14.0 (Stata Corp, Texas, USA). Baseline characteristics will be computed as proportions or median (interquartile range [IQR]), as appropriate, by arm in each of the two stages. Any variables that show imbalances will be adjusted for when testing the null hypothesis of no difference in mean of proportions linking to care or prevention between the SOC and each of the intervention arms.

The main statistical analysis will be for the primary outcome of male partner uptake of HIV testing plus linkage into care or prevention. We will assume that the 2 stages of the trial are independent[36]

and will proceed to carry out a test of the null hypothesis of no difference in effectiveness of each intervention compared to the SOC. All analyses will be by intention-to-treat taking into account the clustered design according to a plan of analysis which will be written. Control for multiple comparisons and multiple stages will use the asd R package[45].

7.2.1. Stage 1 Statistical Analysis

Given the small number (seven) of clusters per arm in the first stage analysis will be by cluster-level summaries using mean of proportion of male partners per clinic day who link to care or prevention in each arm. The proportion of male partners who link into care or prevention will be computed per clinic day for each arm with number of letters returned as the numerator and the number of women present in ANC on enrolment day as denominator. A log transformation of the clinic day proportions will be applied if a positive skew is observed[42]. If baseline imbalance is observed an adjusted analysis, using a two stage process[42] appropriate for a small number of clusters, will be conducted. The mean of clinic day proportions in each of the five intervention arms will be compared to the SOC arm using unpaired t-test. An estimate of the risk ratio (RR) and a 95% CI will also be computed for each comparison.

This analysis involves five comparisons with a single control arm which can lead to higher than the specified family wise error rate (FWER) or significance level. Therefore, we will apply a Dunnett test[46] to the t-statistics generated from the unpaired t-test to control the stage-wise FWER. Final decision-making will compare the Dunnett-corrected P-values to stage 1 FWER of 0.2. Pre-planned decisions and adaptations at the end of stage 1 (Appendix S) are as follows:

a) **Dropping intervention arms for futility**
   Any intervention arm that yields a P-value>0.2 when compared to the SOC arm will be dropped.
b) **Dropping intervention arms due to adverse events**
   Notwithstanding a) above, any intervention arm with cumulative incidence of intimate partner violence i.e. verbal physical violence *related* to study procedures or interventions >1% above that observed in the SOC will be dropped[47].
c) **Maintaining intervention arms due to cost-effectiveness**
   Notwithstanding a) an intervention arm may be maintained after taking into account the cost implications of providing the service associated with that particular intervention
d) **Re-estimation of sample size for the remaining arms**
   Sample size will be re-calculated for the remaining arms based on empirical estimates.

7.2.2. Stage 2 Statistical Analysis

Analysis in this stage constitutes both stage 2 analysis and leads directly to analysis of the whole study. Although sample size will be re-calculated at the end of stage 1, the total number of clinic days per arm is presumed to be small for stage 2, around 12. Since we will assume that each stage is independent, cluster level summaries approach analogous to stage 1 analysis will also be followed in this stage comparing intervention arms that proceed to stage 2 with the SOC arm. Final decisions will then be based on combined P-values from both the first stage and the second stage using the weighted inverse normal (WIN) method[48].

8. Data Safety Monitoring Board (DSMB)

8.1. DSMB

An independent DSMB will be responsible for reviewing the results of the interim analysis (stage 1 analysis results) and will have the final recommendation with regards to dropping an arm for futility, adverse events or cost effectiveness (Appendix S). The board will comprise three members with expert knowledge in HIV self-testing, adaptive trial designs and the local HIV context namely; Prof Frances Cowan (University College London), Dr Patrick Phillips (Medical Research Council, UK) and Prof Victor Mwapasa (College of Medicine), respectively.

The trial will be audited according to standard operating procedures (SOPs) laid down by the MLW Clinical Trial Support Unit (CTSU) internal monitoring committee and the University of Malawi-College of Medicine Research Support Centre (RSC) monitoring process.

9. Ethics, Conflict of Interest, Data Availability and Dissemination

9.1. Ethical Approval

Ethics approval will be sought from the College of Medicine Research Ethics Committee (COMREC), Malawi and the London School of Hygiene and Tropical Medicine Ethics Committee, respectively. Before recruitment to the study, women attending ANC on a day that has been randomised to any of the study arms will be briefed about what this study will entail using group counselling. Study participants will get information about the study and written or witnessed (with thumb print for illiterate participants) consent will be sought before commencing data collection or participation in the allocated trial arm (Appendix F and Q).

As the nature of the intervention is not compatible with written informed consent from male partners, we will request ethics committee waivers from the two ethics committees. We will instead provide information leaflets, a toll-free ("Call Me" SMS number) telephone information service, and directions to clinic-based services. Only authorised personnel will handle the study data with password protection of both the computer and the study database. Final data will be fully anonymised to remove any participant identifying information to uphold confidentiality.

9.2. Conflict of interest

Neither the PI nor any of the collaborators has any conflict of interest.

9.3. Data Availability

The final fully anonymised data from the study will be made publicly available through the LSHTM data repository (http://datacompass.lshtm.ac.uk/.

9.4. Dissemination

Findings from the trial will be disseminated to the Blantyre District Health Office (DHO) and officials in Ministry of Health (MoH) through presentations and final copy of the report. Further local dissemination will be done at the National AIDS Commission (NAC) / College of Medicine (COM) annual dissemination conference. Findings will also be presented at peer-reviewed regional and international conferences. Copies of the final report, published peer-reviewed paper (s) and abstracts will be made available to the COM Library, and to College of Medicine Research Ethics Committee (COMREC).

10. Possible Constraints

A major anticipated constraint is potential for intimate partner violence to women although evidence from studies using other populations and other HTC models suggests this approach is unlikely to increase this problem[10]. For example, in a recent large HIV self-testing study in Blantyre, Malawi increased intimate partner violence was not reported despite an active community liaison system among 27,000 self-testing participants[24]. We will carefully monitor intimate partner violence, and have deliberately listed this as a secondary (safety) outcome. Although it will not be possible for participants and recruiting staff to predict the next-day recruiting arm, the knowledge of FI arms may result in altered decision-making about health care seeking. For example, a woman may choose to postpone her ANC attendance in the hope of being recruited in a FI arm, or indeed want to switch between arms.

There is potential for contamination due to participants in the standard of care arm to benefit from the intervention(s) particularly where participants who receive self-test kits may share with those who did not receive. In order to minimise this problem, we will ask women and their male partners in the intervention arms to bring used / unused self-test kits at follow-up and when they link into the male friendly clinic, respectively. We will also attempt to measure the magnitude of this problem by asking all women and male partners who link into the male friendly clinic in the standard of care if they received self-test kits.

11. Training and Capacity Building

This work is part of my (Augustine Choko) PhD training with LSHTM, funded by the Wellcome Trust, UK. Study personnel for the study will be trained on research ethics, maintaining confidentiality and data collection using tables among other essential skills. Field workers responsible for participant recruitment and study procedures will undergo refresher HIV testing and counselling (HTC) and any other appropriate training where required. All study staff including the principal investigator (PI) will undergo Good Clinical Practice (GCP) training or refresher GCP training as appropriate.

12. Personnel, Materials and Consumables, Equipment, Space and Miscellaneous

12.1. Personnel

Field workers with training and experience in HTC and data collection will be responsible for participant recruitment and follow-up. A field supervisor will supervise the field workers while the PI will provide overall leadership of the study.

12.2. Materials and Consumables

The following materials and consumables will be required for the study

a) OraQuick ADVANCE HIV I/II (OraSure Technologies, Bethlehem, USA) for oral tests
b) Determine 1/2™ (Alere, Waltham, USA) and Uni-Gold™ Recombigen® HIV (Trinity Biotech, Bray, Ireland) for confirmatory HIV testing at the time the male partner links.
c) Writing materials, study materials, visual aides and clip boards will be required during protocol training and other training activities.
d) Printing
e) Vehicle running costs

12.3. Equipment

Three tablets for electronic data capture, lockable filing cabinets for temporary storage of completed consent forms and study tools.

12.4. Space

Space for storing study tools, equipment and consumables as well as private space for conducting study procedures will be required in all recruitment primary health centres.

12.5. Miscellaneous

Airtime for mobile communication between study personnel will be required.

13. Budgetary Estimate

1 Personnel and training	Qty	Unit	Amount	Total (MWK)	GBP
Field worker	4	each/12m	520000	2,080,000	2971
HIV testing & counselling refresher	1	total	1000000	1,000,000	1429
Good Clinical Practice refresher	5	each	150000	750,000	1071
Protocol training	1	total	100000	100,000	143
**2 Implementation costs**					
Participant compensation	900	each	2000	1,800,000	2571
Vehicle hiring costs	12	month	120000	100,000	143
Mobile phone credit	5	each/12m	50000	250,000	357
**3 Equipment & consumables**					
Tablet	4	each	150000	600000	857
Office equipment	3	each	300000	900000	1286
OraQuick test kits	3000	each	1750	5250000	7500
Confirmatory test kits	1200	each	625	750000	1071
Stationary	1	lampsum	1000000	1000000	1429
**Dissemination costs**					
4 National dissemination	2	each	75000	150000	214
5 COMREC fee	1	each	75000	75000	107
Budget total	1			14,805,000	21150
Contribution towards CoM Research Governance	1			1480500	2115
**Grand total**				**16,285,500**	**23,265**

14. References

References

1. World Health Organization. Global Update on the Health Sector Response to HIV, 2000-2015. Geneva, Switzerland: WHO, 2015.

2. Rosen S, Fox MP. Retention in HIV care between testing and treatment in sub-Saharan Africa: a systematic review. PLoS Medicine. 2011;8(7).

3. Naik R, Doherty T, Jackson D, Tabana H, Swanevelder S, Thea DM, et al. Linkage to care following a home-based HIV counselling and testing intervention in rural South Africa. J Int AIDS Soc. 2015;18:19843.

4. Govindasamy D, Kranzer K, van Schaik N, Noubary F, Wood R, Walensky RP, et al. Linkage to HIV, TB and non-communicable disease care from a mobile testing unit in Cape Town, South Africa. PloS One. 2013;8(11).

5. Staveteig S, Wang S, Head SK, Bradley SEK, Nybro E. Demographic Patterns of HIV Testing Uptake in Sub-Saharan Africa. DHS Comparative Reports No. 30. Calverton, Maryland, USA: ICF International: 2013.

6. Honge BL, Jespersen S, Nordentoft PB, Medina C, da Silva D, da Silva ZJ, et al. Loss to follow-up occurs at all stages in the diagnostic and follow-up period among HIV-infected patients in Guinea-Bissau: a 7-year retrospective cohort study. BMJ Open. 2013;3(10).

7. UNAIDS. 90-90-90: An ambitious treatment target to help end the AIDS epidemic. Geneva, Switzerland: 2014.

8. Msuya SE, Mbizvo EM, Hussain A, Uriyo J, Sam NE, Stray-Pedersen B. Low male partner participation in antenatal HIV counselling and testing in northern Tanzania: implications for preventive programs. AIDS Care. 2008;20(6):700-9.

9. Orne-Gliemann J, Balestre E, Tchendjou P, Miric M, Darak S, Butsashvili M, et al. Increasing HIV testing among male partners. AIDS. 2013;27(7):1167-77.

10. Hensen B, Taoka S, Lewis JJ, Weiss HA, Hargreaves J. Systematic review of strategies to increase men's HIV-testing in sub-Saharan Africa. AIDS. 2014;28(14):2133-45.

11. Drake AL, Wagner A, Richardson B, John-Stewart G. Incident HIV during Pregnancy and Postpartum and Risk of Mother-to-Child HIV Transmission: A Systematic Review and Meta-Analysis. PLoS Medicine. 2014;11(2).

12. Mugo NR, Heffron R, Donnell D, Wald A, Were EO, Rees H, et al. Increased risk of HIV-1 transmission in pregnancy: a prospective study among African HIV-1-serodiscordant couples. Aids. 2011;25(15):1887-95.

13. UNAIDS. Counseling and voluntary HIV testing for pregnant women in high HIV prevalence countries. Elements and issues. Geneva, Switzerland: UNAIDS: 2001.

14. Sabapathy K, Van den Bergh R, Fidler S, Hayes R, Ford N. Uptake of home-based voluntary HIV testing in sub-Saharan Africa: a systematic review and meta-analysis. PLoS Medicine. 2012;9(12).

15. Suthar AB, Ford N, Bachanas PJ, Wong VJ, Rajan JS, Saltzman AK, et al. Towards universal voluntary HIV testing and counselling: a systematic review and meta-analysis of community-based approaches. PLoS Medicine. 2013;10(8).

16. Kennedy CE, Fonner VA, Sweat MD, Okero FA, Baggaley R, O'Reilly KR. Provider-initiated HIV testing and counseling in low- and middle-income countries: a systematic review. AIDS Behav. 2013;17(5):1571-90.

17. Hensen B, Baggaley R, Wong VJ, Grabbe KL, Shaffer N, Lo YR, et al. Universal voluntary HIV testing in antenatal care settings: a review of the contribution of provider-initiated testing & counselling. Tropical medicine & international health : TM & IH. 2012;17(1):59-70.

18. Osoti AO, John-Stewart G, Kiarie J, Richardson B, Kinuthia J, Krakowiak D, et al. Home visits during pregnancy enhance male partner HIV counselling and testing in Kenya: a randomized clinical trial. AIDS. 2014;28(1):95-103.

19. Obermeyer CM, Osborn M. The utilization of testing and counseling for HIV: a review of the social and behavioral evidence. Am J Public Health. 2007;97(10):1762-74.

20. Musheke M, Ntalasha H, Gari S, McKenzie O, Bond V, Martin-Hilber A, et al. A systematic review of qualitative findings on factors enabling and deterring uptake of HIV testing in Sub-Saharan Africa. BMC Public Health. 2013;13:220.

21. Chikovore J, Hart G, Kumwenda M, Chipungu GA, Corbett L. 'For a mere cough, men must just chew Conjex, gain strength, and continue working': the provider construction and tuberculosis care-seeking implications in Blantyre, Malawi. Glob Health Action. 2015;8:26292.

22. Kumwenda M, Munthali A, Phiri M, Mwale D, Gutteberg T, MacPherson E, et al. Factors shaping initial decision-making to self-test amongst cohabiting couples in urban Blantyre, Malawi. AIDS Behav. 2014;18 Suppl 4:S396-404.

23. Choko AT, Desmond N, Webb EL, Chavula K, Napierala-Mavedzenge S, Gaydos CA, et al. The uptake and accuracy of oral kits for HIV self-testing in high HIV prevalence setting: a cross-sectional feasibility study in Blantyre, Malawi. PLoS Medicine. 2011;8(10).

24. Choko AT, MacPherson P, Webb EL, Willey BA, Feasy H, Sambakunsi R, et al. Uptake, Accuracy, Safety, and Linkage into Care over Two Years of Promoting Annual Self-Testing for HIV in Blantyre, Malawi: A Community-Based Prospective Study. PLoS Medicine. 2015;12(9).

25. MacPherson P, Lalloo DG, Webb EL, Maheswaran H, Choko AT, Makombe SD, et al. Effect of optional home initiation of HIV care following HIV self-testing on antiretroviral therapy initiation among adults in Malawi: a randomized clinical trial. Journal of the American Medical Association. 2014;312(4):372-9.

26. Thornton RL. The Demand for, and Impact of, Learning HIV Status. The American Economic Review. 2008;98(5):1829-63.

27. Kranzer K, Govindasamy D, van Schaik N, Thebus E, Davies N, Zimmermann M, et al. Incentivized recruitment of a population sample to a mobile HIV testing service increases the yield of newly diagnosed cases, including those in need of antiretroviral therapy. HIV Med. 2012;13(2):132-7.

28. Nglazi MD, van Schaik N, Kranzer K, Lawn SD, Wood R, Bekker LG. An incentivized HIV counseling and testing program targeting hard-to-reach unemployed men in Cape Town, South Africa. Journal of Acquired Immune Deficiency Syndromes. 2012;59(3):e28-34.

29. Car J, Gurol-Urganci I, de Jongh T, Vodopivec-Jamsek V, Atun R. Mobile phone messaging reminders for attendance at healthcare appointments. Cochrane Database Syst Rev. 2012;7:CD007458.

30. de Tolly K, Skinner D, Nembaware V, Benjamin P. Investigation into the use of short message services to expand uptake of human immunodeficiency virus testing, and whether content and dosage have impact. Telemedicine and e-Health. 2012;18(1):18-23.

31. Odeny TA, Bailey RC, Bukusi EA, Simoni JM, Tapia KA, Yuhas K, et al. Text messaging to improve attendance at post-operative clinic visits after adult male circumcision for HIV prevention: a randomized controlled trial. PloS One. 2012;7(9):e43832.

32. Aluisio A, Richardson BA, Bosire R, John-Stewart G, Mbori-Ngacha D, Farquhar C. Male antenatal attendance and HIV testing are associated with decreased infant HIV infection and increased HIV-free survival. Journal of Acquired Immune Deficiency Syndromes. 2011;56(1):76-82.

33. Thirumurthy H, Masters SH, Rao S, Bronson MA, Lanham M, Omanga E, et al. Effect of Providing Conditional Economic Compensation on Uptake of Voluntary Medical Male Circumcision in Kenya A Randomized Clinical Trial. Journal of the American Medical Association. 2014;312(7):703-11.

34. Brown CH, Ten Have TR, Jo B, Dagne G, Wyman PA, Muthen B, et al. Adaptive designs for randomized trials in public health. Annu Rev Public Health. 2009;30:1-25.

35. Royston P, Parmar MK, Qian W. Novel designs for multi-arm clinical trials with survival outcomes with an application in ovarian cancer. Statistics in Medicine. 2003;22(14):2239-56.

36. Bretz F, Schmidli H, Konig F, Racine A, Maurer W. Confirmatory seamless phase II/III clinical trials with hypotheses selection at interim: general concepts. Biometrical journal Biometrische Zeitschrift. 2006;48(4):623-34.

37. Thirumurthy H, Akello I, Murray M, Masters S, Maman S, Omanga E, et al. Acceptability and feasibility of a novel approach to promote HIV testing in sexual and social networks using HIV self-tests. International AIDS Society (IAS) Conference. Vancouver, Canada: Abstract # MOAC 0302LB: 2015.

38. Gouws E, Cuchi P. Focusing the HIV response through estimating the major modes of HIV transmission: a multi-country analysis. Sex Transm Infect. 2012;88 Suppl 2:i76-85.

39. Snow G. blockrand: Randomization for block random clinical trials. R package version 1.3. http://CRAN.R-project.org/package=blockrand 2013.

40. Maheswaran H, Petrou S, MacPherson P, Choko AT, Kumwenda F, Lalloo DG, et al. Cost and quality of life analysis of HIV self-testing and facility-based HIV testing and counselling in Blantyre, Malawi. BMC Med. 2016;14(1):34.

41. Bratton DJ, Phillips PP, Parmar MK. A multi-arm multi-stage clinical trial design for binary outcomes with application to tuberculosis. BMC Medical Research Methodology. 2013;13:139.

42. Hayes JR, Moulton LH. Cluster Randomised Trials: Chapman and Hall/CRC; 2009.

43. Royston P, Barthel FM, Parmar MK, Choodari-Oskooei B, Isham V. Designs for clinical trials with time-to-event outcomes based on stopping guidelines for lack of benefit. Trials. 2011;12:81.

44. R Core team. R: A language and environment for statistical computing. R Foundation for Statistical Computing, Vienna, Austria. URL http://www.R-project.org/. 2015.

45. Parsons N. asd: Simulations for adaptive seamless designs. R package version 2.0. http://CRAN.R-project.org/package=asd. 2013.

46. Dunnett CW. A multiple comparison procedure for comparing Several Treatments with a control. Journal of the American Statistical Association. 1955;50(272):1096-121.

47. Council for International Organizations of Medical Sciences (CIOMS). Benefit-Risk Balance for Marketed Drugs:Evaluating Safety Signals. Geneva: Switzerland: 1998.

48. Bauer P, Kohne K. Evaluation of experiments with adaptive interim analyses. Biometrics. 1994;50(4):1029-41.

15. Appendices

A. Investigators' Short CVs

CVs for all investigators were recently submitted with PASTAL qualitative study.

B. PQ05: Baseline Questionnaire for Women

PQ05: Baseline Questionnaire for Women

Instructions:

i) Prefix each question with B for baseline.
ii) Variable names which appear in the database come after the question number.

Section A: Identifiers and eligibility screen

1. **b01date** Date of interview *[Date]*
2. **b02intid** Interviewer ID *[Numeric 01-10]*
3. **b03ancd** Clinic day # *[Numeric 01-99]*
4. **b04cid** Clinic ID *[Numeric]* ➔ *coded 1=Ndirande; 2=Bangwe; 3=Zingwangwa*
5. **b05arm** Arm *[Numeric 1-6]* ➔ *1=Standard of care; 2=HIV self-test kits only; 3=HIV self-test kits plus low amount financial incentive; 4=HIV self-test kits plus high amount financial incentive; 5=HIV self-test kits plus lottery financial incentive; 6=HIV self-test kits plus phone call reminder.*
6. **b06name** Full name *[String]* ➔ *indicate both first name and surname*
7. **b07pidw** Woman barcode *[Numeric]* ➔ *scan from barcode sheet, place woman barcode in the health passport; place another on the recruitment log.*

Eligibility screen

8. **b08testp** Have you tested for HIV together with your partner in this pregnancy? *[Numeric]* ➔ *1=yes; 0=no*
   Kodi mwayezetsa kachilombo ka HIV ndi wachikondi wanu mu uchembere uno?
9. **b09age** Are you less than 18 years old *[Numeric]* ➔ *1=yes; 0=no*
   Kodi zaka zanu ndi zosaposera 18?
10. **b10status** Is your partner already aware of his HIV positive status and receiving treatment? *[Numeric]* ➔ *1=yes; 0=no*
    Kodi wachikondi wanu akulandira ma ARV panopa?
11. **b11visit** Is this your subsequent ANC visit? *[Numeric]* ➔ *1=yes; 0=no*
    Kodi munabwerako kale ku sikelo mu uchembere umenewu?
12. **b12recru** Were you already recruited in this trial? *[Numeric]* ➔ *1=yes; 0=no*
    Kodi munalowa kale mu kafukufuku ameneyu?
13. **b13resid** Do you live outside urban Blantyre? *[Numeric]* ➔ *1=yes; 0=no*
    Kodi mumakhala kunja kwa mzinda wa Blantyre, mwachitsanzo boma lina kapena kumidzi?
14. **b14pidm** Male partner barcode *[Numeric]* ➔ *scan the barcode on PQ43_male_partner_invitation_letter*

Section B: Woman demographics and antenatal clinic data

15. **b15denom** Total number of women at ANC on that day *[Numeric]* ➔ *automatically filled from a count of completed PQ05 records*
16. **b16dob** What is your date of birth *[date: DD-MM-YYYY]*
    Kodi munabadwa mchaka chanji? Chonde tiwuzeni tsiku, mwezi komanso chaka ngati nkotheka.

17. **b17age** Age *[Numeric] ➔ calculate automatically using DOB and today's date but record if DOB is unknown.*
    Kodi muli ndi zaka zingati?
18. **b18mstat** Marital status *[Numeric] ➔ 1=married; 2=polygamous marriage; 3=living together as if married; 4=never married; 5=widow; 6=Separated; 7=Divorced; 8=married but not living together.*
    Kodi muli pa banja panopa?
19. **b19live** Are you currently living together with your partner? *[Numeric] ➔ Depends on answer to 12) <u>marital status.</u> 1=yes; 0=no.*
    Kodi mumakhala limodzi ndi mwamuna wanu?
20. **b20lit** Can you read a letter or a newspaper? *[Numeric] ➔ 1=yes; 0=no*
    Kodi mumatha kulemba ndi kuwerenga?
21. **b21occ** How can you best describe your main activity or work status? *[Numeric] ➔ 1=Paid employee; 2=Paid domestic worker; 3=Self-employed; 4=Unemployed; 5=Student; 6=Other*
    Kodi mumagwira ntchito yanji?
22. **b22edu** What was the highest level of education that you have completed? *[Numeric] ➔ 0=Never been to school; 1=Primary school; 2=Secondary school no MSCE; 3= Secondary school with MSCE; 4=Higher*
    Kodi maphunziro anu munafika nawo pati?
23. **b23phone** Phone # *[Numeric]*
24. **b24genh** How do you rate your general health? ➔ 1=Uli bwino kwambiri (Excellent); 2=Uli bwino (Good); 3=Choncho (Fair); 4=Siwuli bwino (Poor)
    Kodi mukuwona kuti moyo wanu uli bwanji?
25. **b25test** Have you tested for HIV in this pregnancy? *[Numeric]➔ 1=yes; 0=no*
    Kodi mwayezetsa kachilombo ka HIV mu uchembere uno?
26. **b26selft** Have you ever self-tested for HIV? *[Numeric] ➔ 1=yes; 0=no*
    Kodi munayamba mwaziyezapo nokha kachilombo ka HIV?

Section C: questions about male partner

27. **b27dob** What is your male partner's date of birth *[date: DD-MM-YYYY]*
    Kodi mwamuna wanu anabadwa mchaka chanji? Chonde tiwuzeni tsiku, mwezi komanso chaka ngati nkotheka.
28. **b28age** Male partner's Age *[Numeric] ➔ calculate automatically using DOB and today's date but record if DOB is unknown.*
    Kodi mwamunayu ali ndi zaka zingati?
29. **b29lit** Can your male partner read a letter or a newspaper? *[Numeric] ➔ 1=yes; 0=no*
    Kodi mwamuna wanu amatha kulemba ndi kuwerenga?
30. **b30occ** How can you best describe your male partner's main activity or work status? *[Numeric] ➔ 1=Paid employee; 2=Paid domestic worker; 3=Self-employed; 4=Unemployed; 5=Student; 6=Other*
    Kodi mwamuna wanu amagwira ntchito yanji?

31. **b31edu** What was the highest level of education that your partner completed? *[Numeric]* ➔ *0=Never been to school; 1=Primary school; 2=Secondary school no MSCE; 3= Secondary school with MSCE; 4=Higher*
    Kodi mwamuna wanu maphunziro ake anafika nawo pati?
32. **b32phone** Partner's Phone # *[Numeric]*
33. **b33test** To your knowledge, has your male partner ever been tested for HIV *[Numeric]* ➔ *automatic yes if YES to tested together in this pregnancy; 1=yes; 0=no*
    Malingana ndi momwe mukudziwira, kodi wachikondi wanu anayamba wayezetsapo kachilombo ka HIV?
34. **b34test12m** To your knowledge, has your male partner tested for HIV in the last 12 months *[Numeric]* ➔ *Automatic yes if YES to tested together in this pregnancy. 1=yes; 0=no*
    Malingana ndi momwe mukudziwira, kodi wachikondi wanu wayezetsa kachilombo ka HIV mu miyezi 12 yapitayi?

Section D: Participation in the allocated arm

35. **b35part** Will you participate in the study? *[Numeric]* ➔ *1=yes; 0=no*
    Kodi mukuvomera kutenga nawo mu kafukufukuyu?
36. **b36why** Reasons for not participating in the allocated arm *[Text]*
    Kodi ndi chifukwa chiyani simukufuna kutenga nawo mbali mu kafukufukuyu?

Thank you
Zikomo

We will want to talk to you again in four weeks when you come for your next antenatal visit.
Tikufuna kudzachezanso nanu masabata anayi akubwerawa.

C. PQ06: Follow-up Questionnaire for Women

z **PQ06: Follow-up Questionnaire for Women (ACASI)**

Instructions to database designer

a) Prefix each question with A for ACASI
b) Variable names appear in bold immediately after the question number.
c) Include the test questions in c) below but do not capture the responses into the database table.

Instructions to the field worker

a) Make sure that the participant is oriented in using both the tablet and the ACASI system before leaving them to answer the questions
b) Stick around while maintaining confidentiality so that the participant can call you in case of issues
c) Complete the two test questions below
   I. Did you eat nsima when coming to the clinic? If you ate nsima, press thumbs up 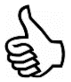 or if you 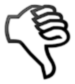 didn't eat nsima, press thumbs down (back end capture as 1=yes; 0=no); if you want the question to repeat, press the white button to repeat the question

   Kodi munadya nsima pobwera kuchipatala?
   [Ngati munadya nsima lero, tobwanyani chala choloza m'mwamba
   Ngati simunadye nsima lero, tobwanyani chala choloza pansi
   Ngati mukufuna kuti funso lifunsidwenso tobwanyani batani loyera
   II. Describe how you moved from home to the clinic. Press the red button once you finish talking; press the white button to repeat the question.
   Fotokozani momwe mwayendera pobwera ku chipatala lero.
   Ngati mukufuna kuti funso lifunsidwenso tobwanyani batani loyera
   [Tobwanyani batani lofiyira mukatha kulankhula]

Section A: Identifiers

Instructions to the field worker

a) Fill in this part before taking leave of the participant
b) Ensure that all identifiers are automatically filled before taking leave of the participant.

1. **a01idate**Date of interview *[Date]*
2. **a02start** Start time *[Time]➔ automatically filled by the tablet*
3. **a03pidw** Woman barcode *[Numeric] ➔ field worker to scan from the woman's health passport*

Section B: Questions related to the activities in the allocated arm, woman specific

4. **f04st** Did you receive self-test kits from anybody? *[Numeric, Y/N]➔ Ask to participants in the SOC arm only*
   Kodi munalandira zipangizo zoziyezera wekha kwa aliyense?

5. **a05let** Did you deliver the letter to your male partner? *[Numeric] ➔ use thumbs up and thumbs down as in c) above all the questions unless stated otherwise*
   Kodi munapereka kalata yomwe munalandira mu kafukufukuyu kwa okondedwa wanu?

[Ngati munapereka, tobwanyani chala choloza m'mwamba
Ngati simunapereke, tobwanyani chala choloza pansi]
Ngati mukufuna kuti funso lifunsidwenso tobwanyani batani loyera

6. **a06kits** Did you deliver self-test kits to your male partner? *[Numeric, Y/N]* ➔ *only to participants in the intervention arms*
   Kodi munapereka zipangizo zozieyezera wekha kwa okondedwa wanu?
   [Ngati munapereka, tobwanyani chala choloza m'mwamba
   Ngati simunapereke, tobwanyani chala choloza pansi]
   Ngati mukufuna kuti funso lifunsidwenso tobwanyani batani loyera
7. **a07ipvl** Did you experience any problems after bringing the letter to your partner? *[Numeric]* ➔ *Y/N*
   Kodi kupereka kalata kuchokera kwa okondedwa wanu kunakubweretserani mavuto ena ali onse?
   [Ngati munakumana ndi mavuto ali onse, tobwanyani chala choloza m'mwamba
   Ngati simunakumane ndi mavuto ali onse, tobwanyani chala choloza pansi]
   Ngati mukufuna kuti funso lifunsidwenso tobwanyani batani loyera
8. **a08ipvk** Did you experience any problems after delivering self-test kits to your partner? *[Numeric, Y/N]* ➔ *Hide this question for participants in the standard of care arm*
   Kodi kupereka zipangizo zoziyezera wekha kwa okondedwa wanu kunakubweretserani mavuto ena ali onse?
   [Ngati munakumana ndi mavuto ali onse, tobwanyani chala choloza m'mwamba
   Ngati simunakumane ndi mavuto ali onse, tobwanyani chala choloza pansi]
   Ngati mukufuna kuti funso lifunsidwenso tobwanyani batani loyera
9. **a09ipv** Please describe any kind of problems you faced? For example: beaten, yelled at, denied sex, marriage break-up, not given money for household use *[Numeric]* ➔ Press the red button once you finish talking.
   Chonde fotokozani mwatsanetsatane mavuto a mtunndu uliwonse omwe munakumana nawo kaya kumenyedwa, kukalipidwa kapena, kukanizidwa za m'banja, kutha kwa banja, kumanidwa ndalama za pakhomo.
   [Tobwanyani batani lofiyira mukatha kulankhula]
   Ngati mukufuna kuti funso lifunsidwenso tobwanyani batani loyera

Section C: Questions related to male partner testing

10. **a10mtest** Did your partner test after you initiated the process? I mean any kind of <u>HIV test</u> *[Numeric, Y'N]*
    Kodi okondedwa wanu anayezetsa kachilombo ka HIV mutamufotokozera? Ndikati kuyezetsa ndikutanthauza kuyezetsa kulikonse kaya ndi ku chipatala, ku malo ena oyezetsera, kapena kuziyeza wekha.
    [Ngati anayezetsa, tobwanyani chala choloza m'mwamba
    Ngati sanayezetse, tobwanyani chala choloza pansi]
    Ngati mukufuna kuti funso lifunsidwenso tobwanyani batani loyera

11. **a11mself** Did male partner self-test? *[Numeric]* ➔ *depends on answer to did male partner test?; hide for participants in standard of care arm.*
    Kodi okondedwa wanu anaziyeza yekha mutamufotokozera?
    [Ngati anaziyeza yekha, tobwanyani chala choloza m'mwamba
    Ngati sanaziyeze yekha, tobwanyani chala choloza pansi]
    Ngati mukufuna kuti funso lifunsidwenso tobwanyani batani loyera
12. **a12self2** Did you self-test together? *[Numeric]* ➔ *depends on answer to did male partner test? ; hide for participants in standard of care arm.*
    Kodi munaziyeza limodzi ndi okondedwa wanu mutamufotokozera?
    [Ngati munaziyeza limodzi, tobwanyani chala choloza m'mwamba
    Ngati simunaziyeze limodzi, tobwanyani chala choloza pansi]
    Ngati mukufuna kuti funso lifunsidwenso tobwanyani batani loyera
13. **a13sharea** Did male partner disclose result if he tested alone *[Numeric]* ➔ *depends on answer to did male partner test? Ask for participants in standard of care arm only.*
    Kodi okondedwa wanu anakuwuzani zotsatira zake ngati anayezetsa payekha?
    [Ngati anakuwuzani, tobwanyani chala choloza m'mwamba
    Ngati sanakuwuzeni, tobwanyani chala choloza pansi]
    Ngati mukufuna kuti funso lifunsidwenso tobwanyani batani loyera
14. **a14shareb** Did male partner disclose result if he self-tested alone *[Numeric]* ➔ *depends on answer to did male partner test? Hide for participants in standard of care arm.*
    Kodi okondedwa wanu anakuwuzani zotsatira zake ngati anaziyeza payekha?
    [Ngati anakuwuzani, tobwanyani chala choloza m'mwamba
    Ngati sanakuwuzeni, tobwanyani chala choloza pansi]
    Ngati mukufuna kuti funso lifunsidwenso tobwanyani batani loyera
15. **a15result** *What was his test result? [Numeric]* ➔ *depends on answer to did male partner test?*
    Kodi zotsatirazo zinali zotani?
    [Ngati anati alibe kachilombo, tobwanyani chala choloza m'mwamba
    Ngati anati alinako kachilombo, tobwanyani chala choloza pansi]
    Ngati mukufuna kuti funso lifunsidwenso tobwanyani batani loyera
16. **a16hard** Did you or your partner experience any difficulties in conducting self-testing? *[Numeric]* ➔ *depends on answer to self-testing*
    Kodi inu kapena okondedwa wanu munakumana ndi vuto lililonse poziyeza nokha?
    [Ngati panalibe vuto lililonse, tobwanyani chala choloza m'mwamba
    Ngati munakumana ndi vuto lililonse, tobwanyani chala choloza pansi]
    Ngati mukufuna kuti funso lifunsidwenso tobwanyani batani loyera
17. **a17link** Did male partner go to the clinic after testing regardless of result? *[Numeric]* ➔ *Y/N, depends on testing questions above*
    Kodi okondedwa wanu anapita ku chipatala potsatira kuyezetsa mutamufotokozera posatengera zotsatira?
    [Ngati anapita, tobwanyani chala choloza m'mwamba
    Ngati sanapite kapena simukudziwa, tobwanyani chala choloza pansi]
    Ngati mukufuna kuti funso lifunsidwenso tobwanyani batani loyera

Thank you for your time, we have to the end of the interview.
Zikomo kwambiri chifukwa choyankha mafunsowa.

D. PQ07: Questionnaire for Men who Link

PQ07: Questionnaire for Men who link to male friendly clinic

Instructions:

iii) Prefix each question with F for Male *Friendly* Clinic.
iv) Variable names which appear in the database come after the question number.
v) The counsellor will scan the barcode on *PQ43_male_partner_invitation_letter and verify that the information available is for the "wife" of the male partner presenting at the male friendly clinic.*

Section A: Identifiers

1. **f01date** Date linked *[Date]*
2. **f02coid** Counsellor ID *[Numeric 01-10]*

Automatically populated upon scanning male partner barcode [3-8]

3. **f03ancd** Clinic day # *[Numeric 01-99]* ➔ *copied from Baseline*
4. **f04cid** Clinic ID *[Numeric]*➔ *coded 1=Ndirande; 2=Bangwe*
5. **f05arm** Arm *[Numeric 1-6]* ➔ *1=Standard of care; 2=HIV self-test kits only; 3=HIV self-test kits plus low amount financial incentive; 4=HIV self-test kits plus high amount financial incentive; 5=HIV self-test kits plus lottery financial incentive; 6=HIV self-test kits plus phone call reminder.*
6. **f06pidw** Woman barcode *[Numeric]*➔ *copied from PQ05 Baseline Form*
7. **f07name** Woman full name ➔*PQ05 Baseline Form*
8. **f08pidm** Male partner barcode *[Numeric]*➔ *copied from PQ05 Baseline Form*

Section B: Demographics

9. **f09dob** What is your date of birth *[date: DD-MM-YYYY]*
   Kodi munabadwa mchaka chanji? Chonde tiwuzeni tsiku, mwezi komanso chaka ngati nkotheka.
10. **f10age** Age *[Numeric]* ➔ *calculate automatically using DOB and today's date but record if DOB is unknown.*
   Kodi muli ndi zaka zingati?
11. **f11mstat** Marital status *[Numeric]* ➔ *1=married; 2=polygamous marriage; 3=living together as if married; 4=never married; 5=widow; 6=Separated; 7=Divorced; 8=married but not living together.*
   Kodi muli pa banja panopa?
12. **f12live** Are you currently living together with your partner? *[Numeric]* ➔ *Depends on answer to 12) <u>marital status.</u> 1=yes; 0=no.*
   Kodi mumakhala limodzi ndi mwamuna wanu?
13. **f13lit** Can you read a letter or a newspaper? *[Numeric]* ➔ *1=yes; 0=no*
   Kodi mumatha kulemba ndi kuwerenga?

14. **f14occ** How can you best describe your main activity or work status? *[Numeric] ➔ 1=Paid employee; 2=Paid domestic worker; 3=Self-employed; 4=Unemployed; 5=Student; 6=Other*
    Kodi mumagwira ntchito yanji?
15. **f15edu** What was the highest level of education that you have completed? *[Numeric] ➔ 0=Never been to school; 1=Primary school; 2=Secondary school no MSCE; 3= Secondary school with MSCE; 4=Higher*
    Kodi maphunziro anu munafika nawo pati?

Section C: HIV testing and follow-on services

16. **f16testb4** Have you ever tested for HIV before the test you just had *[Numeric] ➔ 1=yes; 0=no*
    Kodi munayamba mwayezetsapo kachilombo ka HIV m'mbuyomu kupatula kuyezetsa kwa panopa?
17. **f17test12m** Have you tested for HIV in the last 12 months? *[Numeric] ➔ Depends on 16 above. 1=yes; 0=no*
    Kodi munayezetsa kachilombo ka HIV mu miyezi 12 yapitayi?
18. **f18couple** Came as a couple? *[Numeric] ➔ Don't ask but record accordingly. 1=yes; 0=no; don't ask participant simply record*
19. **f19test2** Did you test together with your partner in this pregnancy? *[Numeric]*
    Kodi munayezetsa limodzi ndi okondedwa wanu mu uchembere wawo wa panopa?

20. **f20test** Did you have an HIV test after your partner informed you?
    Kodi munatengapo gawo pa zoyezetsa HIV wachikondi wanu atakufotokozerani?
21. **f21modet** What was the mode of testing for the test you just had?*[Numeric] ➔ 1=VCT at a testing service or clinic; 2=self-test alone; 3= self-test together; 4=self-test alone followed by self-testing together; 5=self-test alone followed by VCT at a testing service or clinic together; 6=other*
    Kodi kuyezaku kunali mnjira yanji?
22. **f21res** What was the result of your HIV test? *[Numeric] ➔ 1=positive; 0=negative*
    Kodi zakuyezaku zinali zotani?
23. **f23kit** Self-test kit returned? *[Numeric] ➔ 1=yes; 0=no; don't ask participant simply record*
24. **f24rtn** Is the returned **s**elf-test kit (s) used? *[Numeric] ➔1=yes; 0=no; don't ask participant simply record*
25. **f25st** HTC Counsellor re-read of returned used self-test kit *[Numeric] ➔ 1=positive; 0=negative; 2=invalid; don't ask participant simply record*
26. **f26hard** How hard was it for you to do the self-test correctly? *[Numeric]➔ 1=Not at all hard to do the test; 2=somewhat hard to do the test; 3=Very hard to do the test*
    Kunali kovuta bwanji kuti muziyeze nokha molondola?
27. **f27**Did you receive self-test kits from anybody? *[Numeric, Y/N]➔ Ask to participants in the SOC arm only*
    Kodi munalandira zipangizo zoziyezera wekha kwa aliyense?

28. **f28conft** Confirmatory test result *[Numeric]➔ don't ask but record accordingly. 1=positive; 0=negative; don't ask participant simply record*
29. **f29couns** Did the participant receive counselling *[Numeric]➔ 1=yes; 0=no; don't ask participant simply record*

30. **f30cond** Did the participant receive condoms *[Numeric]* ➔ *1=yes; 0=no; don't ask participant simply record*
31. **f31circum** Are you circumcised? *[Numeric]* ➔ *1=yes; 0=no*
    Kodi munapanngitsa mdulidwe wa a bamboo wa ku chipatala?
32. **f32vmmc** Was the participant successfully linked to voluntary medical male circumcision? *[Numeric, Y/N/Refused]* ➔ *1=yes; 0=no, refused; 2=HIV positive; don't ask participant simply record*
33. **f33cd4** CD4 count *[Numeric]*➔*record from result returned*
34. **f34art** Was the participant successfully started on ART or registered for pre-ART *[Numeric]* ➔*1=yes; 0=no, refused; 2=HIV negative; don't ask participant simply record*
35. **f35recom** Would you recommend this *strategy* to other men? *[Numeric]* ➔ *1=definitely yes; 2=not sure; 3=definitely no.*
    Kodi mungawalimbikitse azibambo ena za njira imeneyi?

E. PQ09: Generic Costing Questionnaire

Section A: Identifiers

1 Clinic ID *[Numeric]*

2 Staff ID *[Numeric]*

Section B: Cost items

3 Staff

4 Consumables

5 Equipment

6 Training

7 Capital costs e.g. rent

F. PQ22a: Information Sheet and Consent Women (SOC) -English

Attached

G. PQ22b: Information Sheet and Consent Women (SOC) – Chichewa

Attached

H. PQ23a: Information Sheet and Consent Women (Self-Test Kits only Arm) -English

Attached

I. PQ23b: Information Sheet and Consent Women (Self-Test Kits only Arm) – Chichewa

Attached

J. PQ24a: Information Sheet and Consent Women (Self-Test Kits + a Low Financial Incentive Arm) -English

Attached

K. PQ24b: Information Sheet and Consent Women (Self-Test Kits + a Low Financial Incentive Arm) – Chichewa

Attached

L. PQ25a: Information Sheet and Consent Women (Self-Test Kits + a High Financial Incentive Arm)-English

Attached

M. PQ25b: Information Sheet and Consent Women (Self-Test Kits + a High Financial Incentive Arm) – Chichewa

Attached

N. PQ26a: Information Sheet and Consent Women (Self-Test Kits + a Lottery Financial Incentive Arm) -English

Attached

O. PQ26b: Information Sheet and Consent Women (Self-Test Kits + a Lottery Financial Incentive Arm) – Chichewa

Attached

P. PQ27a: Information Sheet and Consent Women (Self-Test Kits + a Phone Reminder Arm) -English

Attached

Q. PQ27b: Information Sheet and Consent Women (Self-Test Kits + Phone Reminder Arm) – Chichewa

Attached

R. PQ41: Data Management Plan (DMP)

Data Management Plan
for Research Projects

**Project Name**	PASTAL: Partner-provided self-testing and linkage
**Funder**	Wellcome Trust, UK
**Project End Date**	December 2018
**Principal Investigator**	Mr. Augustine T. Choko
**Contact e-mail**	Augustine.Choko@lshtm.ac.uk

Support

Information on writing a Data Management Plan can be found at
http://www.lshtm.ac.uk/research/researchdataman/plan/

One-to-one advice is available through the RDM Support Service
researchdatamanagement@lshtm.ac.uk

DATA DESCRIPTION

1) What data will you collect or create?

Describe the data that you are collecting or creating in your project. Relevant information to provide includes:

- The type of information that will be contained. E.g. MRI scans, interview transcripts, spatial data, etc.
- Methods of capture. E.g. face-to-face interview, web survey, etc.
- Amount of data. E.g. 100 patients will undergo an MRI scan, 500 people will be interviewed.

- Face-to-face interviews with structured paper-based questionnaires with 1,600 participants at baseline and at 4 weeks follow-up
- Audio computer assisted self-interviews (ACASI) using tablets with 1,600 participants at 4 weeks follow-up
- Data will include numerically coded values and an audio-captured field from ACASI

2) Briefly describe the key activities that will be performed on your data, from its creation/capture to its eventual archiving or deletion.

Consider the lifecycle of your research data and the actions that will be performed during that time. For example, data may be captured using a web form, anonymised to remove personal information using software X, cleaned using Tool Y to enable it to be analysed, analysed using software Z, and so on. The lifecycle may be written as text or pictorial form (e.g. a gantt chart).

In addition, it's useful to consider the approximate time period when you will perform each action (e.g. data capture in month 2, data cleansing in month 4, etc.).

- The data captured using paper-based questionnaires will be transported at the end of everyday using project vehicles to Malawi Liverpool Wellcome Trust Clinical Research Programme (MLW) data office for subsequent processing including immediate quality check, scanning, verifying and committing into a MS Access database.
- Tablets running Open Data Kit (ODK) will be used to administer the audio computer assisted self-interview (ACASI) questionnaire. The data on the tablets will be sent to a server AT MLW running MySQL via a secure mobile network with direct download onto the server as a back-up strategy.

3) What data formats or standards will you use to store data produced by your project?

Outline the data formats, encoding standards, or software tools that you will use to create, analyse, or use data. E.g. data will be captured using a MySQL database and analysed using STATA and MS Access.

- Paper-based questionnaires will be scanned using Teleform Optic Mark Reading (OMR) software into an electronic database in MS Access (.mdb). Thereafter, the data will be exported into comma separated values (.csv) for importation into Stata for cleaning and immediate descriptive analysis.
- Data captured using audio computer assisted self-interview (ACASI) will be deposited into a server running MySQL on a daily basis during the study. Thereafter, the data will be exported into comma separated values (.csv) for importation into Stata for cleaning and immediate descriptive analysis.
- The cleaned dataset will be saved in .csv format and will be imported into R software for final analysis.

4) What quality controls and thresholds will you establish to ensure that your data is fit for purpose?

Quality controls may be applied prior, during and following data capture and processing. Possible QC practices include: testing instrumentation to ensure it is correctly calibrated, recording multiple measures, double-entry of information, checking validity of entered values

- All the tools to be used in this trial will be tested in a pilot involving participants from an antenatal clinic that will not be used as a recruitment site.
- All paper-based questionnaires will be checked by a field supervisor at the recruitment site for obvious omissions or errors.
- The completed questionnaires will also be checked before scanning into the Teleform system and during data verification to ensure that correct values are recorded and committed into the study database.
- The participant identifier will be set to be a non-empty field at the design of the questionnaires.
- Inbuilt quality control will be implemented for the data collected using tablets. Skip patterns, range checks and consistency checks will be programmed in open data kit (ODK) to ensure quality data are collected.

5) What documentation or metadata is needed to understand your data?

Describe the documentation or metadata that you will create to enable the data to be understood and used by your future self and others. It is helpful to consider the following questions:

- What information is needed to understand the content and context of its creation?
- What documentation and metadata standards will be used?
- How will potential users find out about your data?

- Each dataset, variable and values will be labelled with separate additional documentation in a data dictionary to be held in MS Word.
- The data will be made publicly available by publication on the Loondon School of Hygiene & Tropical Medicine Data Campus (http://datacompass.lshtm.ac.uk/).

DATA STORAGE AND MANAGEMENT

1) Where will you store data during the project lifetime? (tick one or more)

School PC local drive (drive C: or D:)		**Personal area on School network (drive H:) Yes**		LSHTM Shared Network drive (e.g. I: drive)		**Dedicated server maintained at partner institution Yes**	
LSHTM-based project server		**School laptop or tablet (Yes)**		LSHTM Secure Data Server (for confidential data)		LSHTM Novell Filr	
For-cost cloud service (e.g. Amazon S3)		Free cloud service (e.g. Dropbox, Google Docs)		Portable storage (e.g.USB disk or memory stick)		Other. Please indicate	

Other

2) How will you organise and label your data?

Describe the approach you will take to structure and label your data. E.g. files and folders on a storage device, database tables and labels.

A password-protected folder containing the study database and datasets will be created on the H Drive on the school's network with a similar copy kept on a personal school laptop and on a server at MLW in Malawi.

3) What security measures, if any, will you apply to protect data? (tick one or more)

**Controlled access limited to authorized users only (Yes)**		**Physical security**		**Remove identifiable information (e.g. anonymisation)**	
**Data storage encryption (7 zip)**		**Data transfer encryption**		**Password protection**	
Process on isolated machine in secure room		Secure deletion following analysis			
Avoid use of third party storage, such as Dropbox		Other			

Other

DATA ARCHIVING AND SHARING

1) What data do you need to keep after your project ends and for how long?

The paper-based questionnaires will be kept for a minimum of 10 years before being destroyed.

2) Where will data be kept after your project has finished (tick one or more)

Research data may be submitted to a data repository or data archive, which will handle the process of curation, preservation and sharing on your behalf.

I will keep the data myself		My supervisor will look after the data		It will be looked after by the project team	
Held in the LSHTM Research Data Repository		Held in a LSHTM-maintained project system		Held in a 3rd party data repository. Please specify in Other field	

Other

The data will be kept within the MLW archiving system in Blantyre, Malawi.

3) Can data be made available to anyone? If not state the reason it needs to be restricted and criteria for gaining access.

Can data be made freely available to anyone or do restrictions need to be applied? This question will help you to consider whether access controls need to be applied to limit data access. Potential reasons for restriction include the need to comply with consent agreements, which state:

- Data can only be used by specific users, e.g. researchers working in an academic environment, a specific skill set, etc.
- Data can be analysed only for specific purposes compatible with the consent agreement.

If data does need to be restricted, state the reason and the criteria that users would need to meet to gain access

Yes, the data will be made freely available conformant with the funder requirements (Wellcome Trust, UK).

4) What actions will be performed to prepare your data for access? (tick one or more)

**Removal of personal information (Yes)**		Add synthetic data (e.g. pseudonyms)		Copyright clearance	
**Establish participant consent (Yes)**		Develop an access agreement			

Other

RESOURCING

1) What do you consider to be the primary data management challenges in your project?

What problems or issues do you need to address in your project.

Potential for network failure which may affect daily data transfer from tablets to the server at MLW. However, the field supervisor will ensure that all failed data transfers are done manually by connecting the tablets to the server at MLW.

2) What resources would it be helpful for the School to provide to help deliver your plan?

How can the School help you to manage your data? E.g. training, specific IT Services, etc.

Storage space for large datasets as currently only have 500MB allocated on the H Drive.

S. PQ42: Data Safety Monitoring Board (DSMB) Charter

<table>
<tr><th>Content</th><th>Description</th></tr>
<tr><td colspan="2">1. Introduction</td></tr>
<tr><td>Partner-provided HIV self-testing and linkage (PASTAL).</td><td>The PASTAL Phase II trial is a multi-arm multi-stage adaptive cluster randomised trial (ISRCTN: xxxxxx) funded by Wellcome Trust, UK as a Training Fellowship in Public Health & Tropical Medicine. It will be conducted in urban Blantyre, Malawi and will be based at Malawi Liverpool Wellcome Trust Clinical Research Programme (MLW).</td></tr>
<tr><td>Objectives of trial, including interventions being investigated</td><td>The objectives of PASTAL are as follows:

Primary objective

6) To identify the most promising interventions for increasing both the uptake of HIV testing and linkage into HIV care or prevention among male partners of pregnant women attending antenatal clinic (ANC).

Secondary objectives

7) To identify the most promising interventions for increasing the uptake of HIV testing among male partners of pregnant women attending ANC.
8) To assess the acceptability of partner-provided HIVST-plus, as defined by willingness to deliver HIVST kits to male partners among women attending ANC.
9) To investigate the risk of intimate partner violence among women attending ANC who participate in the study.
10) To provide the cost associated with implementation of the service for each study arm

The interventions will be investigated in two stages aiming to select ≤3 interventions to proceed to a potential Phase III trial (Figure 1). An antenatal clinic day will be randomised to any one of the six arms, with women requested to deliver HIV self-test kits to their male partners in the 5 intervention arms.

Fig. 1: Schema of the Phase II adaptive MAMS CRT and interventions

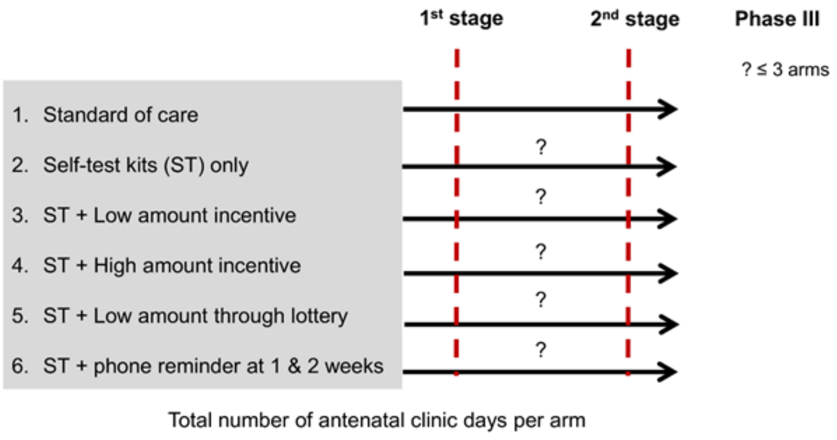
</td></tr>
<tr><td>Outline of scope of charter</td><td>The purpose of this document is to describe the roles and responsibilities of the independent DSMB for the PASTAL trial, including the timing of meetings, methods of providing information</td></tr>
</table>

Content	Description
	to and from the DSMB, frequency and format of meetings and statistical issues.
**2. Roles and responsibilities**	
A broad statement of the aims of the committee	"To protect and serve [trial] participants, particularly women who may be at risk of social harms (especially re: safety) and to assist and advise Principal Investigators so as to protect the validity and credibility of the trial."<br>"To safeguard the interests of trial participants, assess the safety and efficacy of the interventions during the trial, and monitor the overall conduct of the clinical trial."
Terms of reference	The DSMB should receive and review the progress and accruing data of this trial and provide advice on the conduct of the trial to the Trial Investigators.<br>The DSMB should inform the Investigators if, in their view an intervention arm should be dropped after examining evidence relating to:<br>(i) Statistical analysis: an intervention arm should be dropped from the trial because it is no more efficacious compared to the standard of care.<br>(ii) Safety: Notwithstanding (i), an intervention arm should be dropped because of safety concerns by reviewing safety data. a) Comparison of SOC to all intervention arms on the incident cases of intimate partner violence preceding the introduction of interventions; b) Comparison of SOC and each intervention arm separately.<br>(iii) Cost effectiveness: an intervention arm should be dropped or maintained after taking into account the cost implications.
Specific roles of DSMB	Interim review of the trial's progress including updated figures on recruitment, main outcomes and safety data (Figure 2.<br>Specifically, the DSMB will:-<br>• Monitor recruitment figures.<br>• Monitoring evidence for intervention differences in the main efficacy outcome measures.<br>• Monitor evidence for social harms i.e. intimate partner violence (IPV) precipitated by the introduction of the interventions. IPV includes *any* abuse experienced by the woman after delivering HIV self-test kits to the male partner and directly linked to HIV self-testing.<br>• Decide whether to recommend that an intervention arm (s) of the trial be dropped at interim analysis.<br>• Suggest additional data analyses.<br>• Advise on pre-planned adaptations including sample size re-calculation.<br>• Assess the impact and relevance of external evidence
**3. Before or early in the trial**	
Whether the DSMB will have input into the protocol	All potential DSMB members were invited to be part of the DSMB and received a concept note of the trial. The members listed in this Charter fully agreed to be part of the DSMB.

Whether the DSMB will meet before the start of the trial	The DSMB will meet before the trial starts to discuss the protocol, the trial, any analysis plan, future meetings, and to have the opportunity to clarify any aspects with the principal investigators. A "dummy" report with empty shell tables will be presented to familiarise the DSMB members with the format that will be used in the reports.<br><br>The second DSMB meeting will be within a month of completing first stage follow-up to review the accumulating data.<br>All DSBM meetings will be held by Teleconference arranged by the Investigators in Blantyre, Malawi.
**4. Composition**	
Membership and size of the DSMB	The DSMB will comprise three independent members with expert knowledge in HIV self-testing, adaptive trial designs and the local HIV context namely; Prof Frances Cowan (University College London/LSHTM), Dr Patrick Phillips (Medical Research Council, UK) and Prof Victor Mwapasa (College of Medicine), respectively.
The Chair, how they are chosen and the Chair's role. (Likewise, if relevant, the vice-Chairman)	Prof Frances Cowan will chair the DSMB, was chosen by the investigators and has served on DSMBs before. She will facilitate and summarise discussions.
The responsibilities of the DSMB statistician	Dr Patrick Phillips is the DSMB statistician with special expertise in adaptive trial design and provide independent statistical expertise.
The responsibilities of the trial statistician	The trial statistician, Mr. Augustine Choko will produce the report to the DSMB with oversight provided by Dr Katherine Fielding and Prof Nigel Stallard all of whom will participate in DSMB meetings. Mr Choko will guide the DSMB through the report and will take notes.
**5. Relationships**	
Relationships with Principal Investigators	The DSMB will interact directly with the Investigators through Mr Choko (the Principal Investigator).
Clarification of whether the DSMB are advisory (make recommendations) or executive (make decisions)	The DSMB will play an advisory role in this trial and the investigators will make the final decisions based on the recommendations of the DSMB. Recommendations of the DSMB will be communicated in written format to the Investigators through Mr Augustine Choko.
Payments to DSMB members	There will be no payments to the DSMB members other than costs related to logistics of attending the Teleconference meetings, if any.
The need for DSMB members to disclose information about any competing interests	All DSB members will be required to disclose any Competing interests (Annex 1).
**6. Organisation of DMC meetings**	
Expected frequency of DSMB meetings	The trial is expected to complete recruitment and follow-up within a 10 months. Two DSMB meetings are planed; i) to review the trial protocol ii) at 4-5 months to review the data.
Whether meetings will be face-to-	All meetings will be by teleconference.

face or by teleconference

How DMC meetings will be organised, especially regarding open and closed sessions, including who will be present in each session	A mixture of open and closed sessions will be organised as follows:-<br><br>1. Open session: Introduction and any "open" parts of the report attended by Trial Investigators available.<br><br>2. Closed session: DSMB discussion of "closed" parts of the report with Dr Katherine Fielding representing all the Investigators<br><br>and, if necessary,<br><br>3. Open session: Discussion with other attendees on any matters arising from the previous session(s).<br><br>4. Closed session: extra closed session
**7. Trial documentation and procedures to ensure confidentiality and proper communication**	
Intended content of material to be available in open sessions	Open sessions: Accumulating information relating to recruitment, number of women experiencing intimate partner violence and total numbers of events for the primary outcome measure and other outcome measures may be presented, at the discretion of the DSMB.
Intended content of material to be available in closed sessions	Closed sessions: In addition to all the material available in the open session, the closed session material will include efficacy and safety data by trial arm.
Will the DSMB be blinded to the treatment allocation	The DSMB will not be blinded.
Who will see the accumulating data and interim analysis	The following Investigators will see the accumulating data and interim analysis:<br>1. Mr Augustine Choko (Principal Investigator)<br>2. Dr Katherine Fielding<br>3. Prof Liz Corbett<br>4. Prof Nigel Stallard<br><br>DSMB members do **not** have the right to share confidential information with anyone outside the DMC, including the PI.
Who will be responsible for identifying and circulating external evidence (eg from other trials/ systematic reviews)	The Trial Investigators will be responsible for identifying and circulating external evidence (eg from other trials/ systematic reviews).

To whom the DSMB will communicate the decisions/ recommendations that are reached	The DSMB will report its recommendations in writing to the Principal Investigator (Mr Augustine Choko), copied to Dr Katherine Fielding.
Whether reports to the DSMB be available before the meeting or only at/during the meeting	The DSMB report will be made available to the DSMB members via e-mail at least 2 weeks before any meetings.
What will happen to the confidential papers after the meeting	The DSMB members should store the papers safely after each meeting so they may check the next report against them. After the trial is reported, the DSMB members should destroy all interim reports.
**8. Decision making**	
What decisions/recommendations will be open to the DSMB	Possible recommendations could include:-<br>• No action needed, trial continues as planned<br>• Early stopping due to futility, harm (intimate partner violence), or external evidence<br>• Stopping recruitment within a subgroup<br>• Extending recruitment (based on actual control arm response rates being different to predicted rather than on emerging differences) or extending follow-up<br>• Stopping a single arm of a multi-arm trial<br>• Sanctioning and/or proposing protocol changes
The role of formal statistical methods, specifically which methods will be used and whether they will be used as guidelines or rules	We assume that the two trial stages are independent.<br>Total number of clinic days per arm will be provided along with summary statistics and proportions for continuous and categorical baseline variables as appropriate. Total number of male partners linking into care or prevention, and the total number of women reporting intimate partner violence will be computed overall (for the open session) and per arm (for the closed session). Following the intention-to-treat principal, the proportion of male partners linking into care per clinic day per arm will be computed. A cluster-level summary approach to analysis will be followed considering the mall number of clusters per arm for the trial. Proportion estimates from each arm will form a sample from which a mean proportion (standard deviation) will be computed followed by a comparison for each intervention arm and the standard of care (SOC) arm using the unpaired t-test. A log transformation of the clinic day proportions will be applied if a positive skew is observed.<br><br>Analysis will take into account the clustered design with the Dunnett test used to control for multiple comparisons. **As a rule, any intervention arm whose P-value <0.2 will be dropped** from the trial at interim analysis. Randomisation for the second stage will be to those arms that remain in the trial after interim analysis. Thus, the final analysis will use the Weighted Inverse Normal combination method to combine P-values for the two stages of the trial. **As a rule, any intervention arm whose P-value <0.1** in the second stage will not be recommended for a potential Phase III trial.

How decisions or recommendations will be reached within the DSMB	The decision to recommend that an intervention arm be dropped will be reached by:-<br>• Examining the evidence (P-value and confidence intervals) for each intervention compared to the SOC.<br>• Examining the data relating to IPV, specifically focussing on the number of events in the SOC and the rest of the intervention arms; and the SOC and the each intervention arm separately. This examination may reveal two patterns i) suggesting that delivering HIV self-test kits to male partners puts women at high risk of IPV or ii) the use of financial incentives as interventions precipitates IPV in this population.<br>• Considering the points above, an intervention arm deemed less efficacious and also with increased IPV compared to SOC will be recommended to be dropped right away. The DSMB will deliberate and vote for or against dropping an intervention arm which is more efficacious but also has increased IPV compared to the control.<br>• The Chair will summarise discussions and encourage consensus and will break the tie during voting.<br>Details of the voting will not be part of the DSMB meeting to avoid conveying information about the state of the trial data.
When the DSMB is quorate for decision-making	All three members of the DSMB must be present to form a quorum.

**9. Reporting**	
To whom will the DMC report their recommendations/decisions, and in what form	The DSMB will write a formal letter (Annex 2) to Mr Augustine Choko (Principal Investigator) following the DSMB meeting at interim analysis within 3 weeks after the meeting copied to Dr Katherine Fielding.
Whether minutes of the meeting be made and, if so, by whom and where they will be kept	Mr Augustine Choko will take minutes for the open sessions and will file these accordingly. The Chair will appoint a member to take minutes for the closed session and she will sign off any minutes or notes.
What will be done if there is disagreement between the DSMB and the Investigators	In case of disagreement between the DSMB and the trial Investigators a further committee comprising some senior Investigators (Dr Katherine Fielding, Prof Liz Corbett and Prof Nigel Stallard) and an independent external expert may be convened to adjudicate.<br><br>If the DSMB has serious problems or concerns with the Investigators' decision a meeting of these groups should be held. The information to be shown would depend upon the action proposed and the DSMB's concerns. Depending on the reason for the disagreement confidential data will be revealed to all those attending such a meeting. The meeting will be chaired by the external expert.
**10. After the trial**	
Publication of results	At the end of the trial there may be a meeting to allow the DSMB to discuss the final data with principal trial investigators and give advice about data interpretation
The information about the DSMB that will be included in published trial reports	DSMB members will be named and their affiliations listed in the main report, unless they explicitly request otherwise. A brief summary of the timings and conclusions of DSMB meetings will be included in the body of the paper.

Figures and appendices

Figure summarising trial

Annex 1: Competing interest form

Annex 2: Suggested letter from DSMB to Trial Investigators

Figure 2: Randomisation, recruitment, follow-up and outcome evaluation

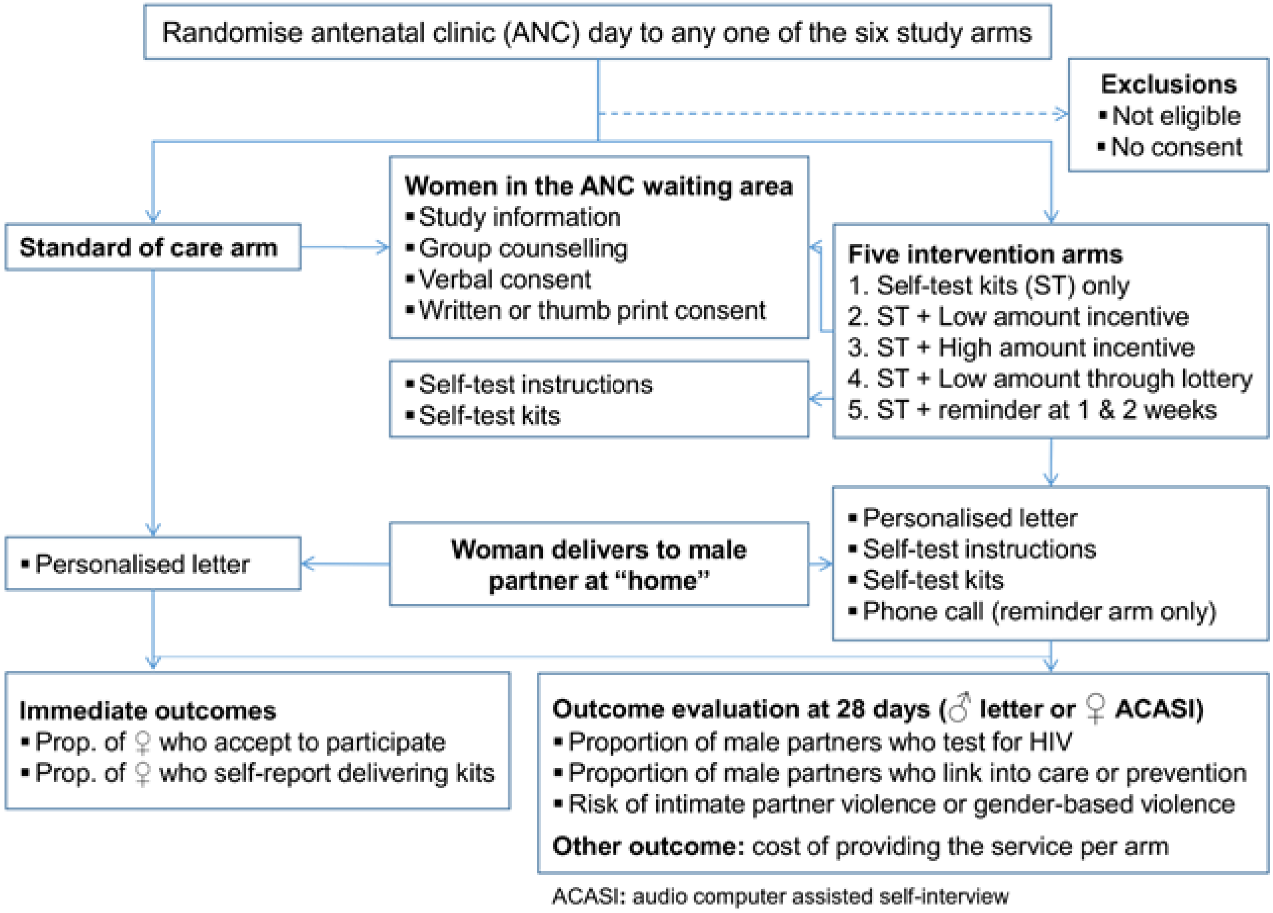

Annex 1: Suggested competing interests form

Potential competing interests of Data Safety Monitoring Board members for *PASTAL*

The avoidance of any perception that members of a DSMB may be biased in some fashion is important for the credibility of the decisions made by the DMC and for the integrity of the trial.

Possible competing interest should be disclosed by informing the trial investigators. In many cases simple disclosure up front should be sufficient. Otherwise, the (potential) DSMB member should remove the conflict or stop participating in the DSMB. Table 1 lists potential competing interests.

Table 1: Potential competing interests

- Stock ownership in any commercial companies involved
- Stock transaction in any commercial company involved (if previously holding stock)
- Consulting arrangements with the sponsor
- Frequent speaking engagements on behalf of the intervention
- Career tied up in a product or technique assessed by trial
- Hands-on participation in the trial
- Involvement in the running of the trial
- Emotional involvement in the trial
- Intellectual conflict eg strong prior belief in the trial’s experimental arm
- Involvement in regulatory issues relevant to the trial procedures
- Investment (financial or intellectual) in competing products
- Involvement in the publication

Please complete the following section and return to the trials office.

☐ **No,** I have no competing interests to declare

☐ **Yes,** I have competing interests to declare (please detail below)

Please provide details of any competing interests:

____

____

Name: ____________________________

Signed: ___________________________ Date: ______________

Annex 2: Suggested report from DSMB to the Trial Investigators where no recommendations are being made

[Insert date]

To: Chair of Trial Steering Committee

Dear [Investigators' representative]

The Data Safety Monitoring Board (DSMB) for the PASTAL trial met on [meeting date] to review its progress and interim accumulating data. [List members] attended the meeting and reviewed the report.

We congratulate the trial organisers and collaborators on the progress and conduct of the trial and the presentation of the data. The trial question remains important and, on the basis of the data reviewed at this stage, we recommend continuation of the trial according to the current version of the protocol [specify protocol version number and date] with no changes.

We shall next review the progress and data [provide approximate timing]

Yours sincerely,

[Name of meeting Chair]
Chair of Data Safety Monitoring Board

On behalf of the DSMB (all members listed below)

DSMB members:
(1) [Insert name and role]
(2) [Insert name and role]
(3) [Insert name and role]

T. Blantyre DHO Letter of Support

Telephone: Blantyre 01875332 / 01877401
Fax: 01872551/01 878 539

Communication should be addressed to:

The District Health Officer

UNITY AND FREEDOM

In reply please quote No. ....

MINISTRY OF HEALTH AND POPULATION
DISTRICT HEALTH OFFICE
P/BAG 66
BLANTYRE
MALAWI

Ref. No. DHO/MED/19 23rd July, 2015

Mr Augustine T. Choko
Malawi Liverpool- Welcome Trust
Clinical Research Programme
P.O Box 30096,
BLANTYRE 3

Dear Sir,

PARTNER-PROVIDED HIV SELF-TESTING AND LINKAGE (PASTAL)

I am pleased to inform you that you have been granted permission to conduct your study entitled "**Investigating interventions to increase uptake of HIV testing and linkage into care or prevention for male partners of pregnant women in antenatal clinics in Blantyre, Malawi**", subject to approval by the College of Medicine Research Ethics Committee.

However, note that the office advises its participation in the study for ownership and use of findings.

Yours sincerely,

Dr. Medson Matchaya
DISTRICT HEALTH OFFICER
