# Supplementary material for: HIV self-testing alone or with additional interventions, including financial incentives, and linkage to care or prevention among male partners of antenatal care clinic attendees in Malawi: An adaptive multi-arm, multi-stage cluster randomised trial
Source: PLoS Med. 2019 Jan 2;16(1):e1002719. doi: 10.1371/journal.pmed.1002719 (PMC6314606; doi:10.1371/journal.pmed.1002719)
Supplement: S2 Text — (PDF) [file pmed.1002719.s007.pdf]

Investigating interventions to increase uptake of HIV testing and linkage into care or prevention for male partners of pregnant women in antenatal clinics in Blantyre, Malawi: an adaptive Phase II multi-arm multi-stage cluster randomised trial (ISRCTN18421340)

Statistical Analysis Plan

(Linked to Protocol v0.5; 03/06/2016)

Principal investigator: Augustine Talumba Choko (LSHTM, MLW)

Co-investigators:

Katherine Fielding	London School of Hygiene & Tropical Medicine (LSHTM)
Elizabeth L Corbett	LSHTM & Malawi Liverpool Wellcome Trust Clinical Research Programme (MLW)
Nicola Desmond	MLW & Liverpool School of Tropical Medicine (LSTM)
Moses Kumwenda	MLW & College of Medicine (COM)
Aurelia Lepine	LSHTM
Hendramoothy Maheswaran	University of Warwick
Nigel Stallard	University of Warwick
Simon Makonbe	HIV Unit, Lilongwe-Malawi

Institutions:

Malawi-Liverpool-Wellcome Clinical Research Programme (MLW), Blantyre, Malawi
Malawi Ministry of Health (MoH), HIV unit (DoHIV/AIDS)
Blantyre District Health Office (DHO), Blantyre, Malawi
Liverpool School of Tropical Medicine (LSTM)
London School of Hygiene and Tropical Medicine (LSHTM), UK

Date: 3rd June 2016

1. Overview of study design, setting and recruitment

The aim of this cluster randomised trial (CRT) is to investigate whether providing HIV self-test kits alone or with an additional intervention through pregnant women who are accessing antenatal care (ANC) for the first time can increase male partner HIV testing and linkage into HIV treatment or prevention services. This is a Phase II trial intended to investigate *safety outcomes*, to provide an estimate of *acceptability* to the woman and *efficacy* relating to uptake of testing and subsequent HIV services by the male partner. The trial involved testing 5 different interventions for improving linkage to the "male friendly" clinic regardless of male partner HIV result including financial incentives and reminders compared to the standard of care (SOC) of giving a personalised letter only to the male partner (annex - Fig. 1). The trial is a multi-arm multi-stage (MAMS) adaptive design with 6 arms (5 intervention and 1 SOC) in the first stage with a planned interim analysis at the end of that stage of recruitment aiming to drop arms and re-calculation of sample size. Enrolment continued in stage two and analysis conducted for participants enrolled into stage two, as well as combing effect estimate across the two stages, where appropriate.

2. Outcomes

Primary outcome

1) The proportion of male partners of ANC attendees who test for HIV and link into HIV care or prevention within 28 days of enrolling the woman.

Secondary outcomes

1) Proportion of male partners of ANC attendees who test for HIV within 28 days.
2) Proportion of women who accept to participate in their allocated study arm.
3) Risk of serious adverse events within 30 days of enrolment associated with each study arm.
4) Total cost of implementing the service per study arm.

<u>Adverse events and safety reporting:</u>

Adverse events will be reported using terms and definitions outlined below

- **Grade 3 events, any of the following within 30 days:**
    - Intimate partner violence that leads to pain, bruising or marks within 24hrs.
    - Threat of life-threatening violence (e.g. statement of intent to kill, mock strangulation, threatened with a knife or gun
    - Physically coercive sex
    - Reports fearing for her life
    - Marriage break

- **Grade 4 events, any of the following within 30 days:**
    - Intimate partner violence leading to hospitalisation or death
    - Suicide or attempted suicide
    - Attack using potentially lethal force (e.g. knife, gun, hammer, kicks to the head)
- **Grade 5, death.**

3. Randomisation

Block randomisation was used to allocate the ANC days across the trial arms in either trial stage. Although recruitment was done from three PHCs no stratification was applied because the PHCs are very similar. The allocation sequence was only communicated to the field workers on the day of recruitment to minimise selection bias.

4. Description of each trial arm

1) Standard of care (SOC): Women will receive personalised letters to give their male partners inviting them to come to the male friendly clinic.
2) Self-test kits only: Women receive a letter and self-test kits to deliver to their male partners.
3) Self-test kits + a $3 financial incentive: Women receive a letter and self-test kits to deliver to their male partners who will get an incentive of $3 conditional on attending the male friendly clinic.
4) Self-test kits + a $10 financial incentive: Women receive a letter and self-test kits to deliver to their male partners who will get an incentive of $10 conditional on attending the male friendly clinic.
5) Self-test kits + lottery incentive: Women receive a letter and self-test kits to deliver to their male partners who will be entered into a lottery with a 10% chance of winning $30 conditional on attending the male friendly clinic.
6) Self-test kits + phone call reminder: Women receive a letter and self-test kits to deliver to their male partners who will receive up to two phone calls to remind them to test and link into male friendly clinic to receive HIV care or HIV prevention services.

5. Sample size justification

Sample size for primary outcome (stage 1)

The choice of 36 clusters provided 80% power to detect a 15% difference from an assumed 25% HIV testing plus linkage for male partners in the SOC arm, assuming a 0.1 value for the coefficient of variation (k).

6. Trial profile

A trial profile figure based on the extension of the CONSORT for cluster randomised trials[1] (annex-Figure 2) will be produced for each of the two trial stages illustrating the following:

i. Number of ANC days (clusters) in each randomisation group
    a. Total number of women and men across clusters
    b. Median number of women and men per clusters by trial arm
ii. Number of clusters lost to follow up
iii. Number of clusters and individuals analysed for each trial outcome, by arm

7. Baseline enrolment characteristics

Analyses will be done in R [2] and Stata 14.0 (Stata Corp, Texas, USA). Baseline characteristics will be computed as proportions or median (interquartile range [IQR]), as appropriate, by arm in each of the two stages of the trial. We will examine baseline characteristics of men as reported by the woman (annex - Table 1 & 2), as well as baseline characteristics of women (annex - Table 3 & 4).

Individual level characteristics

- Age
- Literacy
- Highest level of education
- Occupation
- Marital status
- HIV testing history

8. Statistical analysis of primary outcome

We will assume that the two stages of the trial are independent [3] and will proceed to carry out a test of the null hypothesis of no difference in effectiveness of each intervention compared to the SOC. We will do this by analysing data from the first stage first followed by interim decisions to drop arms; then we will conduct and analyse data from the second stage (no overlap of participants from the first stage). The primary outcome analysis of the whole trial will thus combine p-values and estimates from the each separate stage[3]. The p-values from either stage will be combined using the weighted inverse normal (WIN) method [4] for arms that are not dropped at interim. A weighted average of the log (risk ratio [RR]) will be computed for each intervention arm vs SOC, for the whole trial using estimates from each trial stage (Table 5). The estimates will be weighted using the inverse of the variance. All analyses will be by

intention-to-treat taking as the denominator the number of women who were eligible and take into account the clustered design.

In each cluster the proportion of male partners with the primary outcome will be calculated as:

$$\frac{\text{number of male partners who have an HIV test} + \text{linked to male friendly clinic}}{\text{number of women recruited in that antenatal clinic day}}$$

The distribution of these cluster-level estimates will be examined graphically by trial arm in each stage and a logarithm of the cluster-level summaries applied prior to further analysis if the distributions are right-skewed[5]. The geometric mean of clinic day proportions in each of the five intervention arms will be compared to the SOC arm using unpaired t-test [5]. An estimate of the RR and a 95% CI will also be computed for each comparison by dividing the geometric mean of proportions in each intervention arm and the geometric mean of proportions in the SOC arm[5]. Any variables that show imbalances will be adjusted for using a 2-stage analytical approach for both stages. First, a logistic regression model will be fitted to obtain a residual for each cluster; then these residuals will be analysed in place of the observed estimates.

This analysis involves more than two comparisons with a single control arm which can lead to higher than the specified family wise error rate (FWER) or significance level. Therefore, a Dunnett test [6] will be applied to the t-statistics generated from the unpaired t-test to control the stage-wise FWER. Final decision-making at interim analysis will compare the Dunnett-corrected p-values to stage 1 FWER of 0.2. Since the two stages are assumed to be independent, cluster level summaries approach analogous to stage 1 analysis will also be followed in stage 2 comparing intervention arms that proceed to stage 2 with the SOC arm.

Adaptations at interim analysis (end of stage 1)

Interim analysis at the end of stage 1 will assess whether any of the five intervention arms should be dropped as recommended by an independent data monitoring and safety board (DSMB) based on a 3-part criteria. First, an arm whose statistical comparison to the SOC arm yields a p-value>0.2 will be considered for dropping for futility. Second, any intervention arm with *high* incidence of SAEs i.e. grade 3, 4 or 5 compared to SOC will be considered for dropping. It is the discretion of the DSMB to decide based on absolute number of SAEs in each intervention trial arm whether they are high or not. Such an

observation and recommendation will then be shared with the investigators who will make the final decision. Thirdly, an arm may be maintained after taking into account the costs associated with providing the service in light of the p-value from statistical analysis. For this cost analysis, we will provide the DSMB estimates of the incremental cost per male partner tested, and incremental cost per male HIV positive identified through the intervention arms in comparison to the SOC arm. The investigators will access the first stage data only after the last follow-up visit for participants has occurred in order to perform interim analysis.

Descriptive analysis

We will explore intervention effects on the absolute number of male partners who started ART or got linked to VMMC by trial arm and trial stage (annex – Table 6). The components of the primary outcome will further be broken down into a) men who completed linkage i.e. started ART or got circumcised b) men whose linkage was incomplete: linked to ART or VMMC but did not start ART or were not circumcised.

We will explore if results differ by the self-reported HIV status of the woman; age of the man, recent HIV testing (<12 months); education level.

An estimate of the between-cluster variation, *k*, will be provided for each stage.

9. Analysis of secondary outcomes

The four secondary outcomes are listed as follows:

i) Proportion of male partners who test for HIV within 28 days (as reported by the woman). This will be measured through audio computer assisted self-interview (ACASI) with the woman after her enrolment in the trial. The proportion and 95% Binomial Exact confidence interval (CI) of male partners reported to have had a test following the woman reenrolment will be computed using the number of women eligible at enrolment as the denominator.
ii) Proportion of women who accept to participate in their allocated trial arm. Measured by acceptance of the woman to continue participation in the allotted trial arm after knowing the activities in that particular arm. The proportion and 95% Binomial Exact CI of women who continue participation will be computed using the number of women eligible as the denominator, and shown by trial arm (Table 8).
iii) Risk of serious adverse events (SAEs) by males and females in the study; and the total cost of implementing each trial arm **(see Section 10).**

10. Safety analysis

The total number of participants experiencing serious adverse events (SAEs) will be given as absolute numbers per arm. We expect these to be small hence no formal comparisons to the SOC will be made (annex- Table 7).

11. Sources of bias and contamination

Potential sources of bias which will be examined include:

- Contamination between clusters: it is possible for women or men from the SOC to benefit from the interventions such as by getting self-test kits. We will examine the proportion of participants in the SOC arm who report having received a self-test kit.
- We will check the fidelity of the phone call reminder by examining the proportion of participants who were successfully reached by the study staff

Annex: Figures and Tables

Figure 1: Trial schema

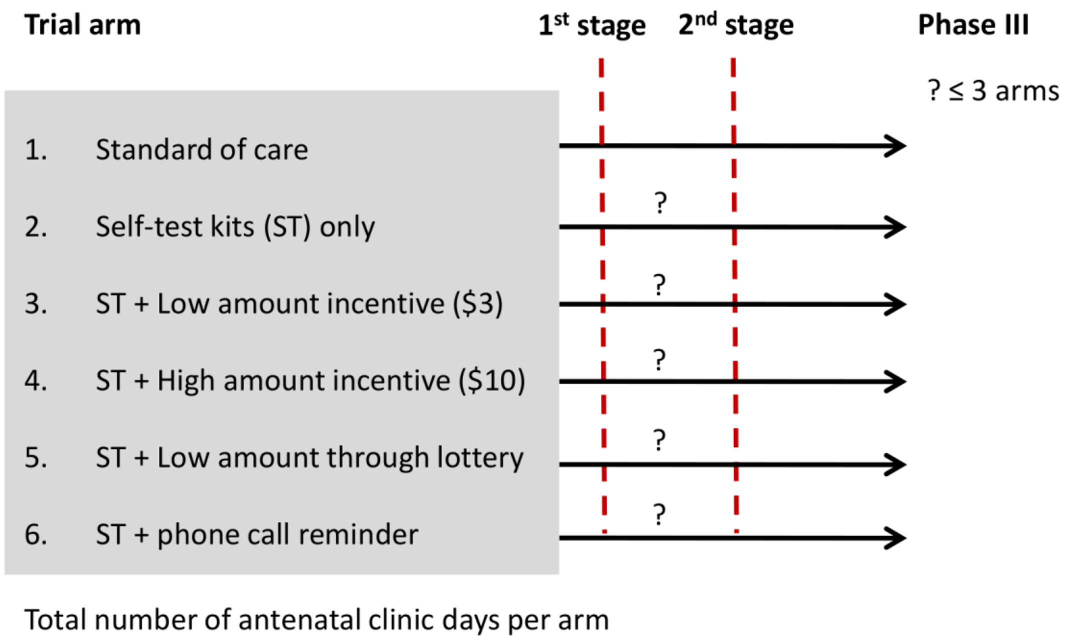

Figure 2. Stage 1 & 2 trial profile

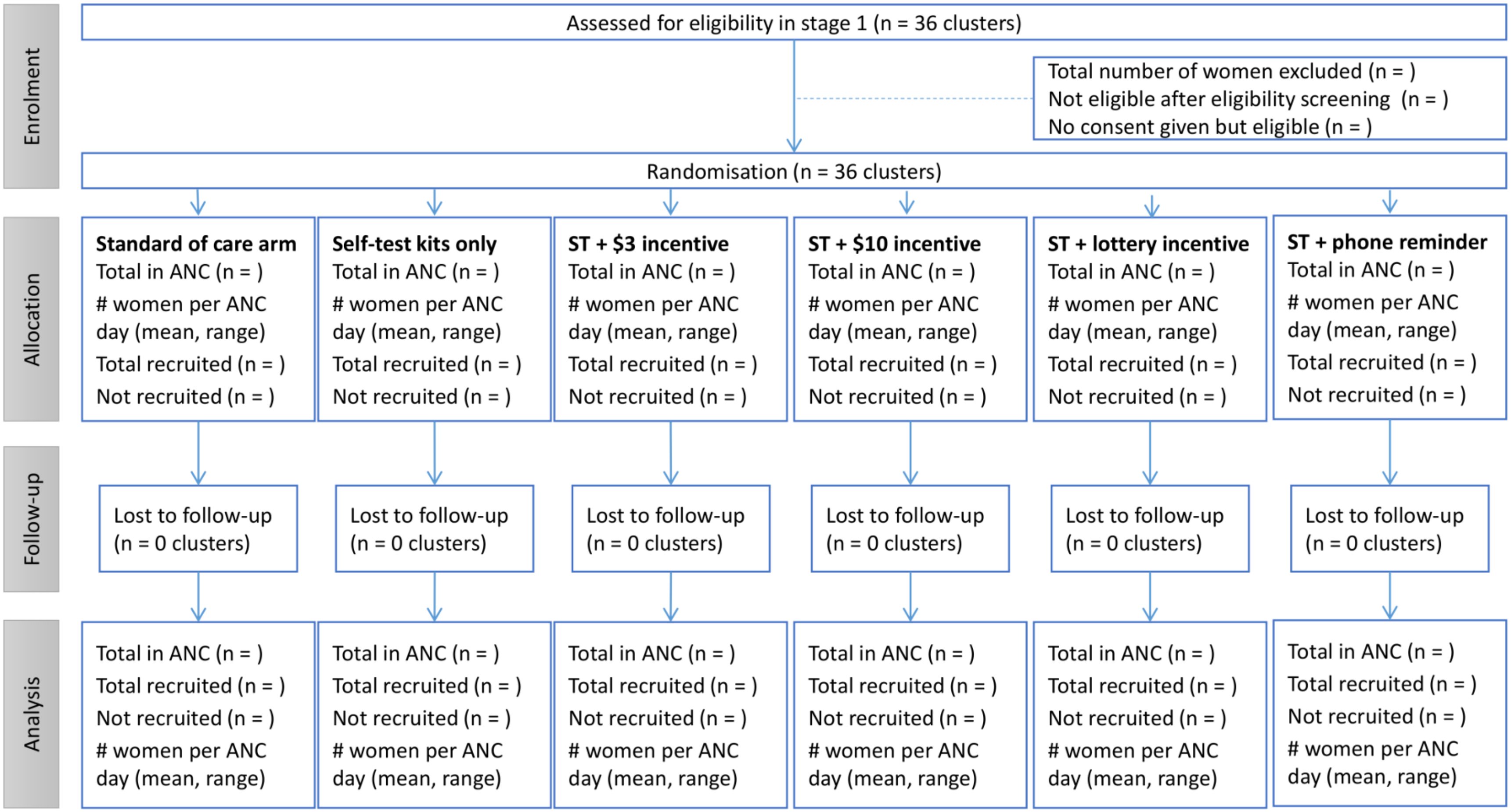

Table 1 & 2: Stage 1 & 2 baseline characteristics of men as reported by the woman (N = XXXX)

		Trial arm					
**Variable**	**Characteristic**	**SOC**	**ST**	**ST + $3**	**ST + $10**	**ST + Lottery**	**ST + reminder**
Number responded	n						
Age (years)	Mean (sd)						
Age group	18-19						
	(20,30]						
	(30,60]						
Able to read and write	No						
Education	Never been to school						
	Primary						
	Secondary						
	Higher						
Occupation	Paid employee						
	Self-employed						
	Unemployed						
Ever tested for HIV	No						
Tested for HIV in the last 12m	No						
Recruitment PHC	Ndirande						
	Bangwe						
	Zingwangwa						

SOC: standard of care; ST: self-test kits; sd: standard deviation; MSCE: Malawi school certificate of education (4 years); PHC: primary health clinic

Table 3 & 4: Stage 1 & 2 baseline characteristics of women (N = XXXX)

		Trial arm					
**Variable**	**Characteristic**	**SOC**	**ST**	**ST + $3**	**ST + $10**	**ST + Lottery**	**ST + reminder**
Number responded	n						
Age (years)	Mean (sd)						
Age group	18-19						
	(20,30]						
	(30,60]						
Able to read and write	No						
Education	Never been to school						
	Primary						
	Secondary						
	Higher						
Occupation	Paid employee						
	Self-employed						
	Unemployed						
Ever tested for HIV	No						
Tested for HIV in the last 12m	No						
Recruitment PHC	Ndirande						
	Bangwe						
	Zingwangwa						

SOC: standard of care; ST: self-test kits; sd: standard deviation; MSCE: Malawi school certificate of education (4 years); PHC: primary health clinic

Table 5: Intervention effects by trial arm and trial stage

	**Trial arm**					
	**SOC**	**ST only**	**ST + $3**	**ST + $10**	**ST + Lottery†**	**ST + Reminder‡**
**First stage**						
Eligible						
Outcome*						
Proportion**						
RR	1					
95% CI						
p-value	NA					
**Second stage**						
Eligible					Dropped	
Outcome*						
Proportion**						
RR						
95% CI						
p-value						
**First stage + second stage**						
RR#						
95% CI						
p-value§						

SOC: standard of care; ST: self-test; RR: risk ratio
† 10% chance of winning $3 times number of men achieving the outcome
‡ phone call
* Evidence of testing and linked to clinic within 28 days regardless of test result
** Geometric mean of the cluster proportions
# Inverse variance weighted risk ratio
§ Using weighted inverse normal method

Table 6: Starting HIV treatment or linkage to male circumcision by trial arm across the two stages

	**SOC**		**ST only**		**ST + $3**		**ST + $10**		**ST + lottery**		**ST + reminder**		**Overall**	
	n	%	n	%	n	%	n	%	n	%	n	%	n	%
**Confirmatory testing**														
HIV positive														
HIV negative														
**Total linked**														
**Completed linkage**														
ART														
VMMC														
**Linkage referred (incomplete)**														
ART														
VMMC														
**No ART or VMMC indicated**														
HIV-ve already circumcised														
HIV+ve already on ART														
Referred for ART or VMMC														
**Of total eligible**														

SOC: standard of care; ST: self-test kits; VMMC: voluntary male medical circumcision

Table 7: Adverse events and ACASI participation in women by trial arm and trial stage

	Trial arm						
	**SOC**	**ST**	**ST+$3**	**ST+$10**	**ST+Lottery**	**ST+reminder**	**Total**
**First stage**							
Total number eligible for ACASI*							
Number interviewed at follow-up							
% of those eligible for ACASI							
Reported on an adverse event							
Through ACASI							
Direct report							
Through male friendly clinic							
**Second stage**							
Total number eligible for ACASI*							
Number interviewed at follow-up							
% of those eligible for ACASI							
Reported on an adverse event							
Through ACASI							
Direct report							
Through male friendly clinic							

ACASI: audio computer-assisted self-interview; SOC: standard of care; ST: self-test kits

* Excluding number of women who discontinued after giving consent

Table 8: Stage 1 & 2 women participation by trial arm stage

		Trial arm					
	**Total**	**SOC**	**ST**	ST + $3	**ST + $10**	**ST + Lottery**	**ST + reminder**
**Stage 1**							
Eligible	**1084**	198	187	146	216	155	182
Consented	**1084**	198	187	146	216	155	182
Discontinued	**77**						
by trial arm	**7.1%**	14 (7.1%)	7 (3.7%)	8 (5.5%)	19 (8.8%)	13 (8.4%)	16 (8.8%)
**Stage 2**							
Eligible							
Consented							
Discontinued							
by trial arm							

SOC: standard of care; ST: self-test kits

References

1. Campbell MK, Piaggio G, Elbourne DR, Altman DG, Group C. Consort 2010 statement: extension to cluster randomised trials. Bmj. 2012;345:e5661.
2. R Core team. R: A language and environment for statistical computing. R Foundation for Statistical Computing, Vienna, Austria. URL http://www.R-project.org/. 2015.
3. Bretz F, Schmidli H, Konig F, Racine A, Maurer W. Confirmatory seamless phase II/III clinical trials with hypotheses selection at interim: general concepts. Biometrical journal Biometrische Zeitschrift. 2006;48(4):623-34.
4. Bauer P, Kohne K. Evaluation of experiments with adaptive interim analyses. Biometrics. 1994;50(4):1029-41.
5. Hayes JR, Moulton LH. Cluster Randomised Trials: Chapman and Hall/CRC; 2009.
6. Dunnett CW. A multiple comparison procedure for comparing Several Treatments with a control. Journal of the American Statistical Association. 1955;50(272):1096-121.
